# The daily resolved temperature dependence and structure of planktonic foraminifera blooms

**DOI:** 10.1038/s41598-020-74342-z

**Published:** 2020-10-15

**Authors:** N. Chernihovsky, A. Almogi-Labin, S. S. Kienast, A. Torfstein

**Affiliations:** 1 The Fredy & Nadine Herrmann Institute of Earth Sciences, The Hebrew University of Jerusalem, 9190401 Jerusalem, Israel; 2 The Interuniversity Institute for Marine Sciences, 8810302 Eilat, Israel; 3 Geological Survey of Israel, 32 Yesha’ayahu Leibowitz Street, 9692100 Jerusalem, Israel; 4 Department of Oceanography, Dalhousie University, Halifax, NS B3H 4R2 Canada

**Keywords:** Ecology, Ecology, Ocean sciences

## Abstract

Planktonic foraminifera (PF) life cycles are highly sensitive to marine conditions, which are evolving rapidly due to anthropogenic climate change. Even though PF shells in the sedimentary record serve as prominent proxies of past ocean conditions, very little is still known about their life cycles, particularly in oligotrophic environments. Here, we present a full annual record of PF fluxes (> 63 µm) from the oligotrophic Gulf of Aqaba, northern Red Sea, sampled at daily timescales during 2015–2016 using an automated time-series sediment trap. These results are coupled with daily surface chlorophyll-*a* concentrations, sea surface temperatures (SSTs), particulate organic carbon and bulk fluxes, together with monthly resolved vertical profiles of chlorophyll-*a*, temperatures and nutrient concentrations. The annual cycle of PF fluxes is controlled by SST changes that drive water column mixing and changes in food availability. PF species flux patterns and succession dynamics vary throughout the year, displaying large variability on previously undocumented daily-weekly timescales, and are not synchronized with lunar periodicity. On daily timescales, spring blooms show a complex structure and interplay between SSTs, chlorophyll-*a* surface concentrations and PF fluxes. These events deliver about a third of the total annual PF flux over a period of several weeks.

## Introduction

Sedimentary Planktonic Foraminifera (PF) shell assemblages and their geochemical composition are widely utilized as proxies of past oceanic conditions such as temperature, salinity, ocean stratification, atmospheric CO_2_ concentrations and biological productivity^1–3^. Considering that PF comprise a major fraction of deep-sea carbonate sediments (~ 32–80%), understanding their life cycles and delivery dynamics to the sediments is critical for understanding the history of the marine carbon cycle and hence, the mechanisms regulating atmospheric CO_2_ levels^4,5^. Indeed, anthropogenic CO_2_ emissions are driving ocean acidification that has been suggested to impose reduced PF calcification rates, resulting in thinner shells and lower PF shell weights, and shifts in their assemblage composition, in pace with the warming trends of the last 150 years^6–9^. This warrants the question of whether and how these organisms will be further affected by ongoing changes in environmental conditions. Hence, there is an acute need to improve our understanding of PF species assemblages and life cycles since accurate reconstruction of past oceanic conditions, largely depends on a better understanding of their living conditions.

PF species life cycles, and hence their vertical shell fluxes, have been associated with the influence of Sea Surface Temperature (SST) and salinity^1,5,10^, phytoplankton productivity and food availability^2,11,12^, nutrient availability and water column configuration^13,14^. A large fraction of the annual vertical flux is known to occur during seasonal phytoplankton blooms, which are constrained to regions with well-defined hydrographic seasonal cycles typically characterizing mid and high latitudes^15^. Yet the temporal dynamics of such blooms occurring at sub-seasonal timescales remain poorly constrained, limiting our understanding of their dynamics or for example, the correlation to lunar cycles^16^. Available observational records in open ocean waters are usually limited to several weeks or months due to the difficulties in maintaining continuous monitoring and sampling schemes, which are typically based on sediment trap moorings that provide a critical link between upper water column processes affecting the living PF community, and their fossil counterparts preserved in the sedimentary record^16–24^.

The Gulf of Aqaba (GOA), northern Red Sea, is a deep and narrow oligotrophic basin, whose properties resemble those of oligotrophic oceanic gyre-center environments^25,26^. A unique deep water column vertical mixing to 300–850 m depth occurs during late fall-winter leading to spring phytoplanktonic blooms. The proximity to land makes it a very accessible study site resulting in a wealth of studies of its biological, chemical and oceanographic dynamics^25–37^.

Recently, a sediment trap -based monthly record of PF vertical fluxes^38^ demonstrated that PF vertical fluxes follow a strong seasonal pattern controlled by nutrient availability, in itself modulated by a wintertime decrease in SSTs that drives deep vertical mixing (> 300 m) and causes nutrient entrainment into the euphotic zone, supporting an increase in primary production. The same mechanism was described in the oligotrophic Sargasso Sea^13^, where the depth of the mixed layer regulates the PF flux by controlling the abundance and timing of their food availability. Likewise, PF succession at the eastern Northern Atlantic is stimulated by redistribution of chlorophyll-*a* (Chl-*a*) and admixing of nutrient-enriched waters into the mixed layer, in concert with seasonal hydrographic changes^15^.

We report here for the first time a record of PF and marine particulate (including organic carbon) fluxes in the GOA at a ~ 1–3 day resolution over more than a full annual cycle, based on a continuous deployment of a sediment trap mooring between March 2015 and April 2016. These observations are part of the Red Sea Dust, Marine Particulates and Seawater Time series (REDMAST) campaign^39^, and are coupled with daily surface Chl-*a* concentrations, SSTs and monthly vertical profiles of Chl-*a*, temperature and nutrient concentrations. The findings are used to characterize the annual (and sub-annual) cycle patterns of PF fluxes and evaluate their driving mechanisms and environmental implications, including an evaluation of lunar-phase synchronized reproduction cycles. We further describe and analyze the internal structure and timescale dynamics of PF shell fluxes during the spring blooms and quantify their contribution to the cumulative annual cycle. Species assemblages are reported and used to configure the short-term succession dynamics of the PF species.

### Gulf of Aqaba

The GOA is a deep, elongated and narrow (average depth of 800 m and maximum depth of 1830 m) marginal water body separated from the Red Sea by the shallow sill of the Straits of Tiran (~ 252 m) (Supplementary Fig. S1). Regional climate is hyper-arid (precipitation < 20 mm/year) and surrounding watersheds are extremely small, resulting in a negligible influx of freshwater and terrigenous material and nutrients into the GOA. The water column is warm throughout the year with seasonal fluctuations in water temperature (20–29 °C), salinity (40.3–41.6 ‰), Chl-*a* (0.03–1.95 µg L^−1^), primary production (0.1–1.9 mg C m^−2^ d^−1^). Oxygen saturation is close to 100% throughout the entire water column^30,40^.

**Figure 1:**
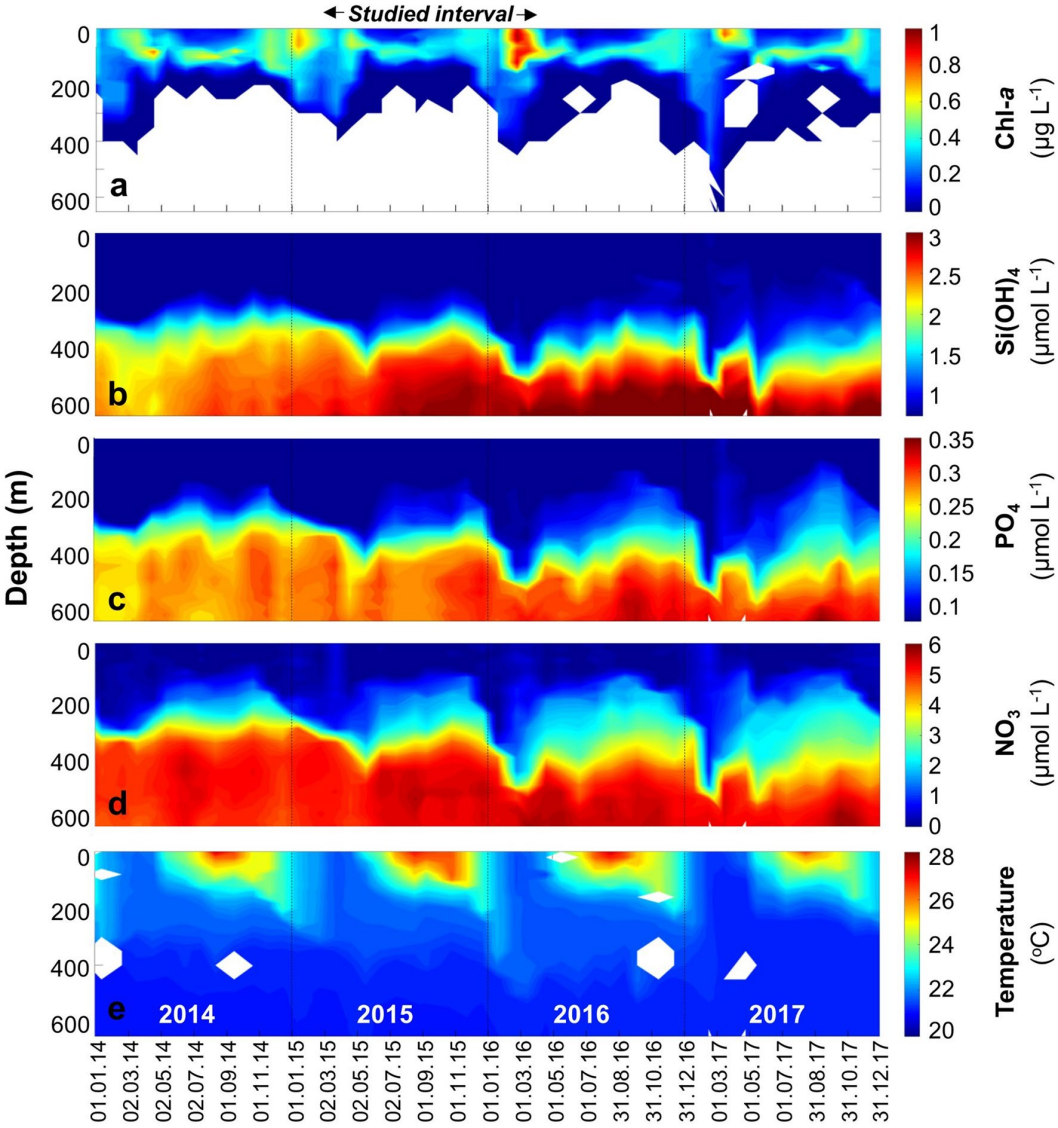
Gulf of Aqaba (GOA) oceanographic configuration. Chl-*a* concentrations (**a**), macro nutrients Si(OH)_4_ (**b**), PO_4_ (**c**), NO_3_ (**d**) and Temperature (**e**) in the GOA during 2014–2017. Data provided by the National Monitoring Program^40^. This study focuses on the planktonic foraminifera dynamics between March 2015 and April 2016. White areas in (**a**) mark no measurable Chl-*a*, and in (**e**) sampling gaps.

During summer (May–September), the water column is stratified, and the surface water layers are nutrient-depleted (Fig. 1). During late autumn, winter and early spring months however, the thermocline gradually deepens and deep convective mixing persists for several months, often reaching 600 m or more, enriching the surface water with deep, nutrient-rich waters. The depth of mixing modulates the seasonal particulate flux, nutrient concentrations and primary production, as well as the dynamics of the recurring spring bloom in which microphytoplankton biomass and particulate organic carbon standing stocks peak^33^.

The phytoplankton community is dominated by *Synechococcus* and eukaryote cells (dinoflagellates and diatoms) during winter and spring blooms, while *Prochlorococcus* is dominant in nutrient-depleted summer-stratified waters^31,32^. Planktonic foraminifera fluxes peak during late winter with the two most dominant species being *Turborotalita clarkei* and *Globigerinoides ruber* (white)^38^. It should be noted that this species assemblage is very different from the one documented during the early 1970s, when the most abundant species was *Trilobatus sacculifer*^37^, currently not found in the GOA^38^.

## Results

### Particulate fluxes

**Figure 2:**
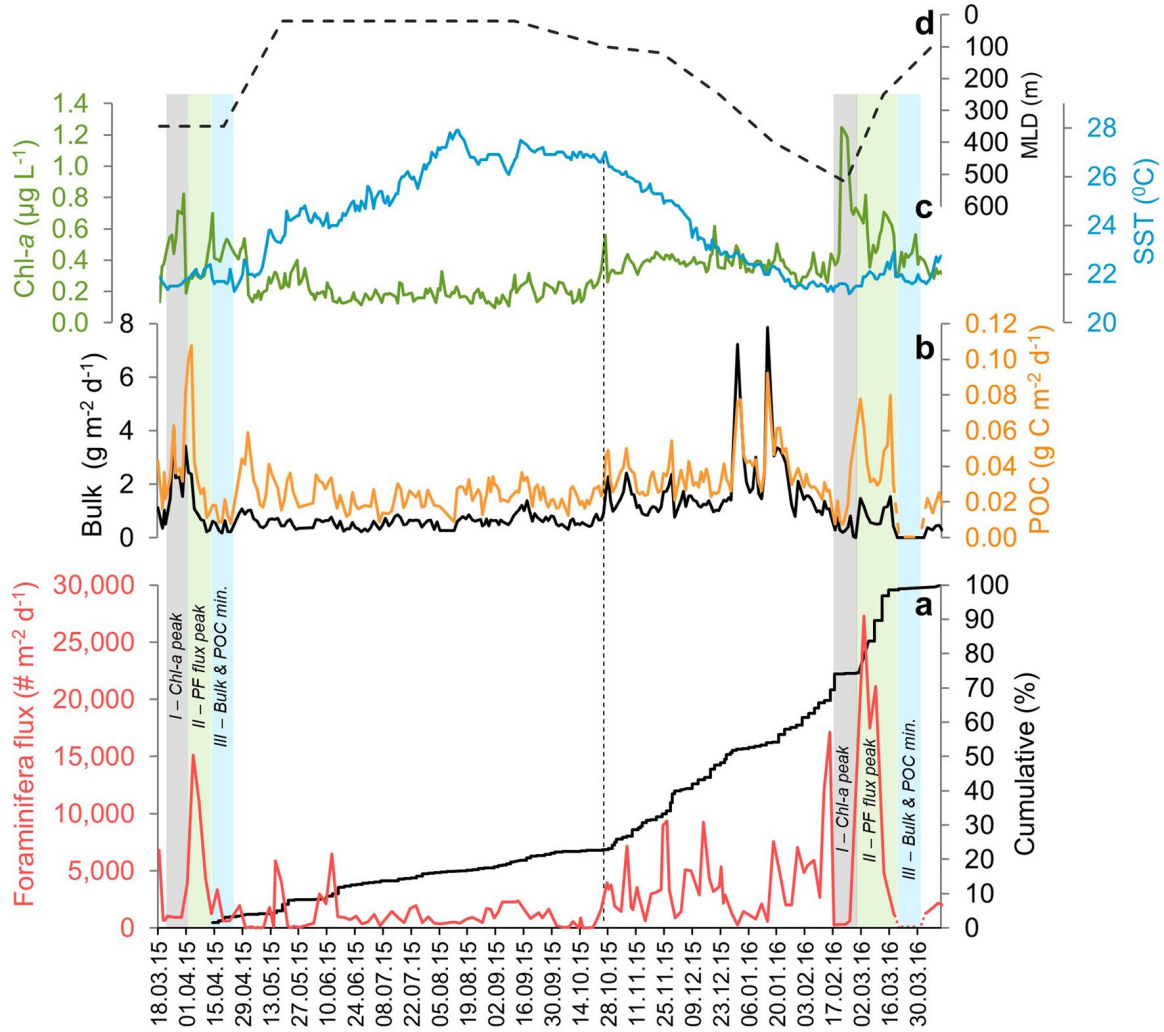
GOA dynamics between March 2015 and April 2016. (**a**) daily timescale fluxes of planktonic foraminifera (PF, red) and their annual cumulative flux (black, starting on April 10th 2015), (**b**) Bulk and particulate organic carbon (POC) fluxes, (**c**) daily surface Chl-*a* concentrations (green) and SSTs (blue), (**d**) mixed layer depth calculated from monthly temperature depth profiles at *station A* (Supplementary Fig. S1). A vertical dashed curve marks the onset of winter cooling associated with an increase in Chl-*a*, bulk, POC and PF fluxes. The structure of the two spring bloom events captured in this study is highlighted by vertical rectangles, marking *phases I, II* and *III* of the events (see text for details).

**Figure 3:**
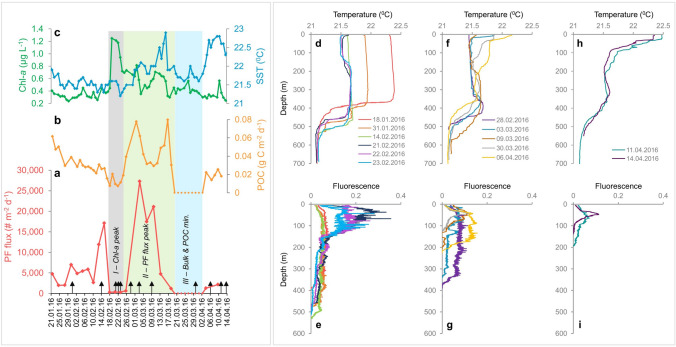
Left box: daily spring bloom dynamics between January 21st and April 14th, 2016. (**a**) planktonic foraminifera fluxes (PF, red), (**b**) particulate organic carbon (POC) fluxes and (**c**) surface Chl-*a* concentrations (green) and SSTs (blue). Vertical rectangles mark *phases I*, *II* and *III* of the spring bloom events (see text for details). Black arrows along the lower abscissa mark the timing of water column depth profiles presented in panels (**d**–**i**). Right box: Temperature- (**d**, **f**, **h**) and fluorescence- (**e**, **g**, **i**) depth profiles between January and April 2016. The mixed layer temperature drop during January culminates in a spike in fluorescence values at intermediate depths between February 21–23, corresponding with high surface Chl-*a* concentrations (**d**, **e**) while the downward flux of planktonic foraminifera crashed (referred to here as *phase I* of the spring bloom). Subsequently, gradual warming of the surface waters while slight cooling occurs at intermediate depths (**f**), corresponds with a drop in the total integrated fluorescence values (**g**) (integrated values not shown), similarly reflected by a drop in Chl-*a* concentrations (**c**), when PF fluxes reach their annual high (**a**, **f**, **g**; *phase II*). Thereafter, a second minimum in PF and POC fluxes corresponds with ongoing warming of the mixed layer and diminishing fluorescence values (**h**, **i**).

A total of 262 sediment trap samples were collected and processed for the time period between March 17th, 2015 and April 10th, 2016 (Supplementary Table S1). Of these, PF were picked from 138 samples (> 63 µm) with individual trapping times between 12 and 40 hours, averaging at 33 hours (Supplementary Table S2). Samples collected between 18.03.16 and 03.04.16 were not picked for PF due to extremely low bulk fluxes (Figs. 2, 3) and are considered to be devoid of PF shells.

The bulk particulate flux ranges between baseline values ca. 0.2–0.3 g m^−2^ d^−1^ and peak values of up to 8 g m^−2^ d^−1^ observed during discrete winter events. Particulate Organic Carbon (POC) fluxes generally follow the bulk flux pattern, with baseline values ca. 0.01 g C m^−2^ d^−1^ and maximal annual values of up to 0.11 g C m^−2^ d^−1^. Annually, the baseline of total PF shell fluxes is < 2000 # m^−2^ d^−1^ and is perturbed during discrete events when fluxes reach an order of magnitude higher (Fig. 2). These perturbations can be divided into two groups, the first consisting of intermediate peaks (< 10,000 # m^−2^ d^−1^) during early winter and late spring months, and the second, occurs during late winter and early spring months (February to April) and is characterized by extremely high values (> 15,000 # m^−2^ d^−1^). Summer PF fluxes (June to October) display continuously low baseline values.

### Planktonic foraminifera species assemblages

**Figure 4:**
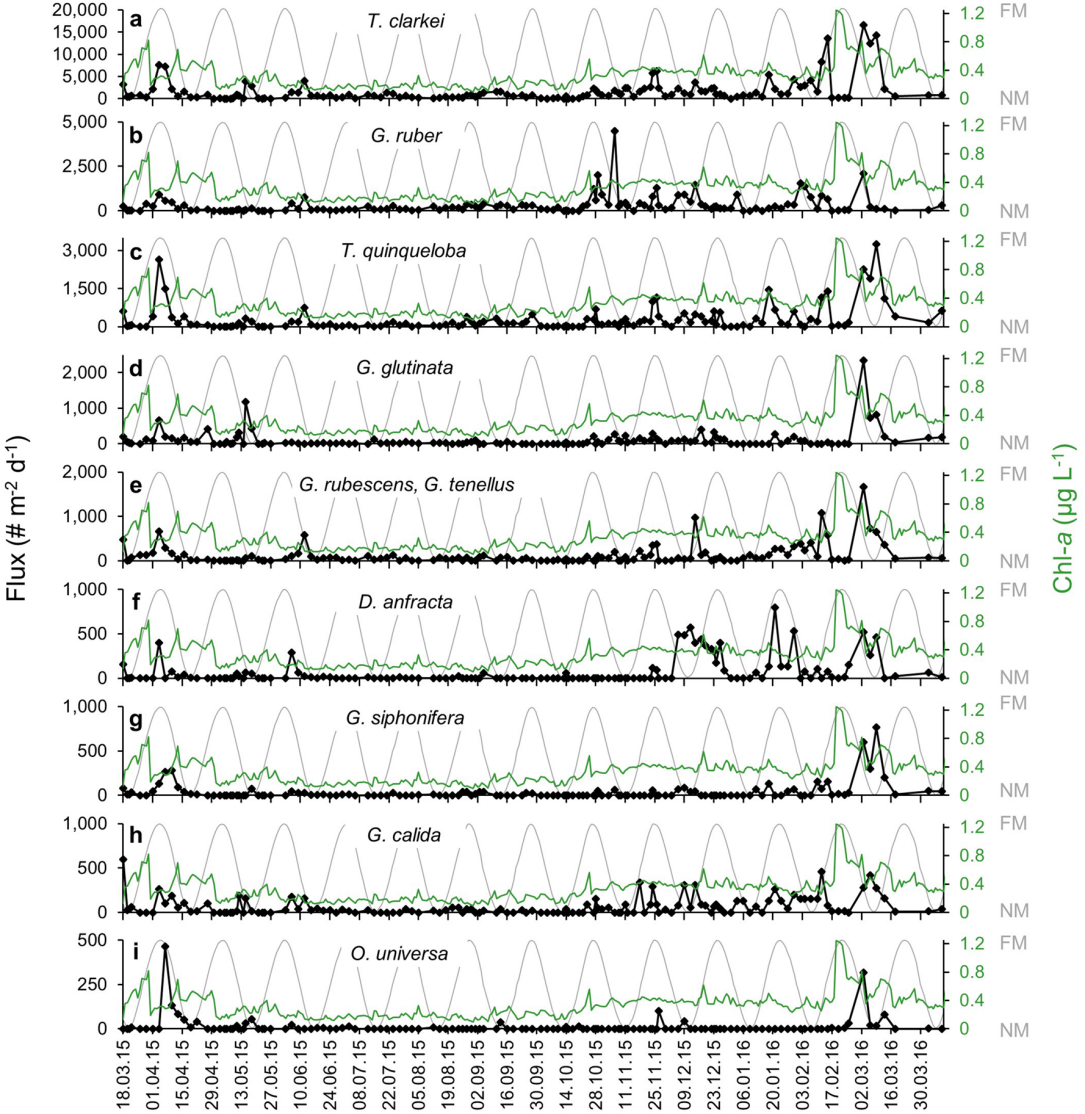
Planktonic foraminifera species fluxes: (**a**) *T. clarkei*, (**b**) *G. ruber*, (**c**) *T. quinqueloba*, (**d**) *G. glutinata*, (**e**) *G. rubescens* & *G. tenellus*, (**f**) *D. anfracta*, (**g**) *G. siphonifera*, (**h**) *G. calida*, and (**i**) *O. universa.* Each panel includes coeval Chl-*a* surface concentrations (green curve) and lunar illumination (grey curve).

The three most abundant species identified in this study are *Turborotalita clarkei*, *Globigerinoides ruber* (white) and *Turborotalita quinqueloba*, comprising of 60, 11 and 10% of the total PF assemblages, respectively (Fig. 4, Supplementary Table S2). Additional species identified include *Globigerinita glutinata* (3.8%), *Dentigloborotalia anfracta* (2.6%), *Globigerinella calida* (2.4%), *Globigerinella siphonifera* (1.2%) and *Orbulina universa* (0.4%). Small shells of the species *Globoturborotalita rubescens* and *Globoturborotalita tenellus* were not distinguishable at the species level, and were therefore classified together as "*G. rubescens* + *G. tenellus*" (4.4%). Broken shells (> 50% shell), containing proloculus and very small unidentified neanic stages were classified as "Unidentified" (4.4%).

### Planktonic foraminifera species fluxes

PF fluxes can be categorized according to two typical temporal patterns: (a) A gradual increase in the magnitude of pulses throughout the winter, reaching an annual peak value during the spring coeval with maximal Chl-*a* concentrations (e.g., *T. clarkei*, *T. quinqueloba*, *G. rubescens* and *G. tenellus*, Fig. 4). Among this group, some species display distinct short-term pulses during the spring bloom (i.e., *G. glutinata*, *G. siphonifera* and *O. universa*). (b) Species whose fluxes do not peak during spring, but rather display fluctuating values throughout winter and spring, as well as  during the fall (i.e., *G. ruber* and *D. anfracta*, Fig. 4).

**Figure 5:**
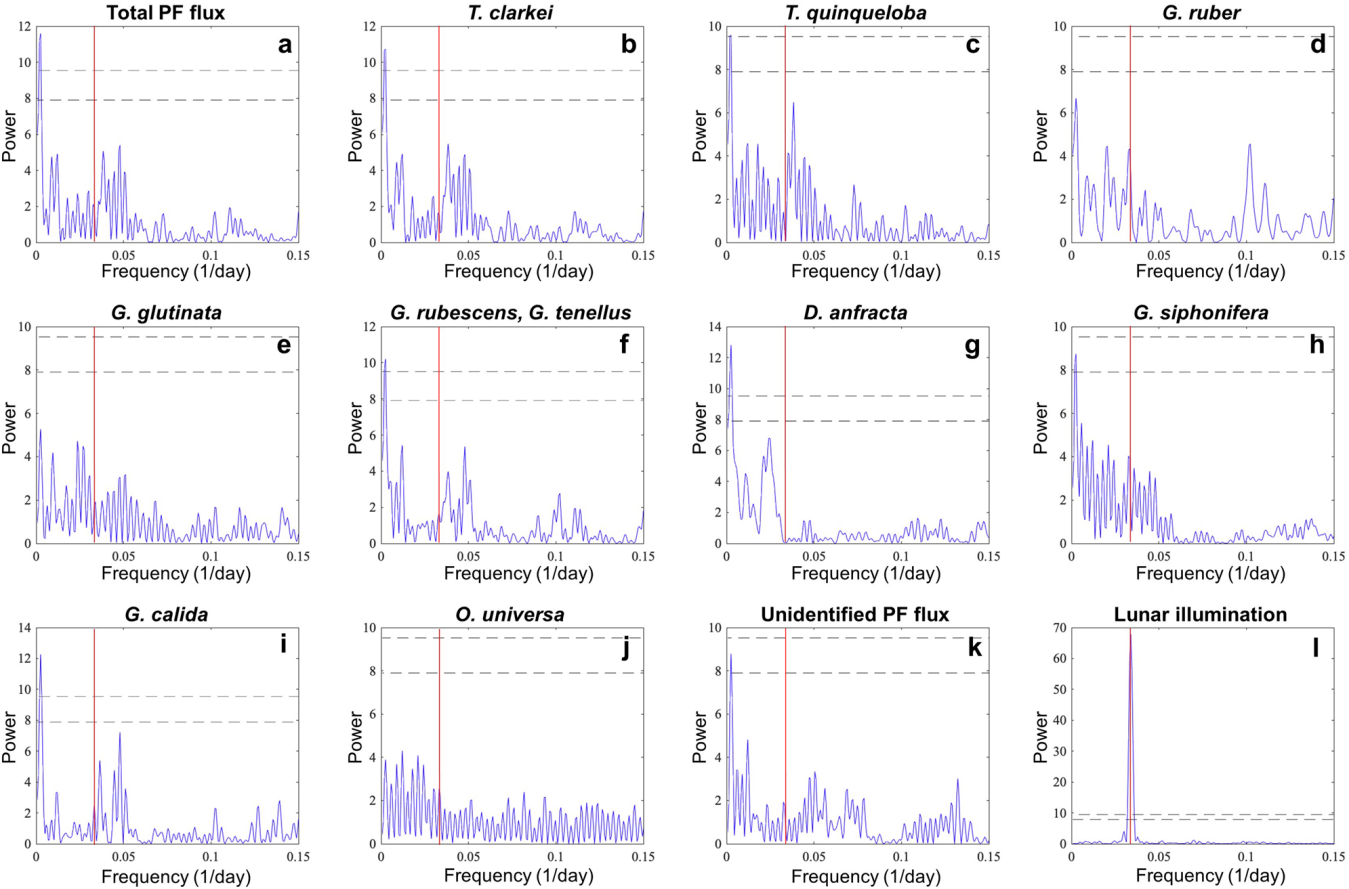
Lomb-Scargle periodograms of: (**a**) Total PF fluxes, (**b**) *T. clarkei*, (**c**) *T. quinqueloba,* (**d**) *G. ruber*, (**e**) *G. glutinata,* (**f**) *G. rubescens* & *G. tenellus*, (**g**) *D. anfracta,* (**h**) *G. siphonifera,* (**i**) *G. calida,* (**j**) *O. universa,* (**k**) Unidentified PF flux*,* and (**l**) lunar illumination*.* The two horizontal lower and upper dashed curves mark a 95% and 99% detection probability, respectively. The red vertical curve marks a 29.5 day cycle that corresponds to lunar periodicity. See more details in text.

Interestingly, although all PF species display repeated short flux pulses, their recurrence does not appear to be associated with the lunar phase nor is it associated with any other significant periodicity (Fig. 5, Supplementary Figs. S2, S3). It should be noted that *G. ruber* displays a minor peak at 29.5 days (0.0339 day^-1^), which becomes statistically significant when a spectral analysis is performed on an interpolated (continuous) data set, however, this is considered to be an artifact of the data interpolation (see details in *Methods*). Furthermore, examination of lunar-phase-related local maxima peaks demonstrated that none of the species displayed an increased number of peaks, or an increase in peak fluxes (orange histogram bars and black circles, respectively, in Supplementary Fig. S2) during the third quarter phase, shortly after the full moon. By contrast, it appears that both peak numbers and their fluxes display a drop shortly before the full moon (second quarter phase) in several species (e.g., *T. clarkei*, *T. quinqueloba*, *G. ruber*, *D. anfracta*, *G. siphonifera*).

**Figure 6:**
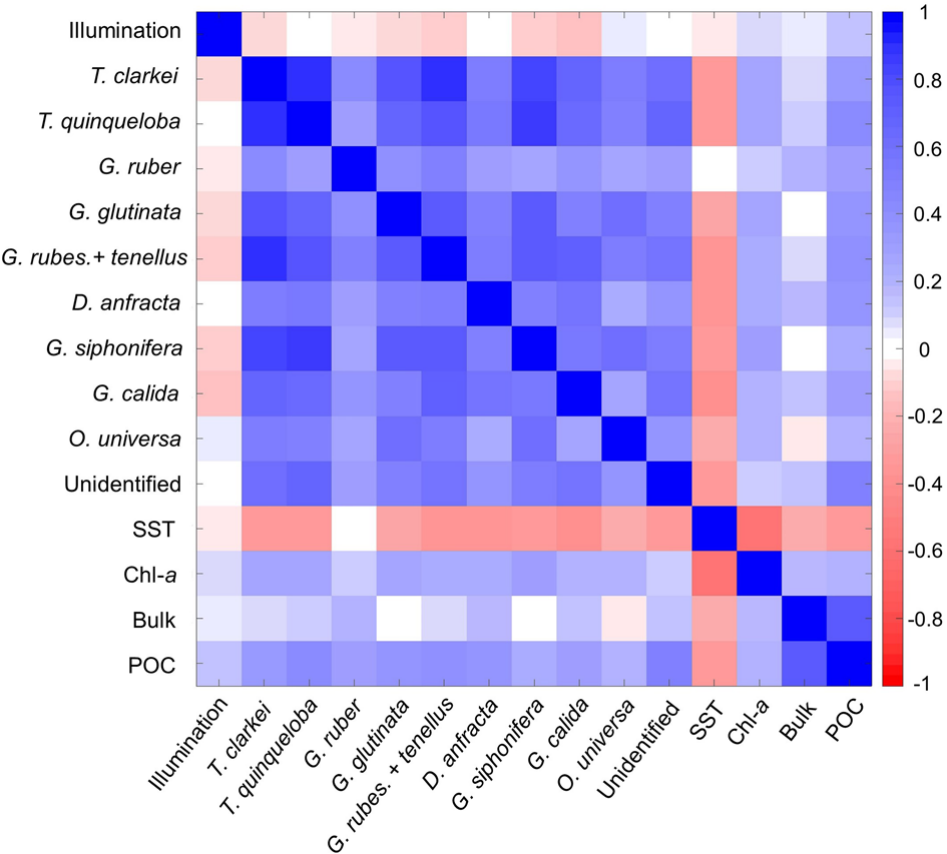
Correlation matrix of planktonic foraminifera species, lunar illumination, SST, Chl-*a*, and particulate bulk and POC fluxes. Correlation coefficients are presented in Supplementary Table S3.

The correlation coefficients between the different PF species fluxes demonstrate that to a first order they vary together and are positively correlated (Fig. 6, Supplementary Table S3), although *G. ruber* and to a lesser extent *D. anfracta,* display a relatively weak correlation with the rest of the species. In terms of relation to environmental parameters, PF fluxes display a weak negative correlation with SSTs, which is not significantly altered when considering time lags (Supplementary Figs. S4–S7). The bulk particulate flux displays a weak and inconsistent correlation with PF fluxes, while the correlation between POC and PF fluxes peaks when considering a time lag of a few days. This time lag is short (1–4 days) for most species, with *G. siphonifera* and *G. calida* displaying a smeared peak lasting up to ~ 7–8 days of an inferred time lag (Supplementary Fig. S6).

## Discussion

### Temporal patterns

Planktonic foraminifera shell fluxes display a distinct seasonal pattern with minimum values during summer and maximum values during the late winter and spring (Fig. 2). These seasonal patterns however, are driven by large variations in fluxes over very short (daily-weekly) time scales.

Throughout the summer, as SSTs reach 28 °C and the water column is thermally stratified, PF fluxes, together with the bulk particulate and POC fluxes are low. The transition to a cooling mode takes place during the fall. During the 2015 fall this transition takes place across a single day, October 25th (vertical dashed line in Fig. 2). On that same day, Chl-*a* surface concentrations display their first major spike of the season, coinciding with a brief rise in SST and a subsequent drop that continues throughout the winter. Following this transition, the first intermediate level spike of PF and POC fluxes is observed on October 27th. From that point onward, with declining SSTs, PF fluxes display intermediate peaks not exceeding 10,000 # m^-2^ d^-1^. An overall similar pattern is observed for bulk and POC fluxes, although both latter display their highest annual values during two brief events in January 2016. The patterns of these two events are not reflected by the PF flux or the Chl-*a* surface concentrations, and their details will be discussed in a separate publication.

The magnitude of the PF spring fluxes, as well as surface Chl-*a* concentrations, are correlated with the mixing depth (Fig. 2). Generally, the convective mixing in the GOA reaches ~ 300–400 m although it has also reached as deep as > 800 m during unusually cold winters^29,41^. Peak PF shell fluxes during the 2016 mixing event, when the mixed layer depth (MLD) reached 525 m, was 50% higher than those observed during 2015, when the MLD reached 350 m.

### Spring blooms

Two spring bloom events were recorded during the course of this study, March 2015 and February–March 2016. While the two events display an overall similar structure of a coeval Chl-*a* peak and PF flux minima (*phase I*), followed by a drop in Chl-*a* and peak in PF fluxes (*phase II*) and a final crash in bulk, POC and PF fluxes (*phase III*) (Fig. 3), the 2016 event is more pronounced in terms of overall fluxes as well as Chl-*a* concentrations (Fig. 2). Given that most of the time period covered by this study leads up to the 2016 spring bloom, providing an appropriate reference to understanding its dynamics, we focus most of our attention on this event. While ‘*spring blooms*’ are typically associated with phytoplankton blooms that are reflected by Chl-*a* peaks, we identified a precursor peak in PF fluxes (February 15th), prior to the Chl-*a* peak (February 19th). This PF peak could be considered to be part of the sequence of intermediate winter PF flux peaks, though a similar feature is observed before the 2015 spring bloom, suggesting that this is part of a systematic pattern. We postulate this peak is associated with short-term mortality due to predation by different succession peaks of larger zooplankton in the water column. Interestingly, when Chl-*a* displays its highest values recorded throughout this study (1.25 µg L^-1^; February 19th), PF (and POC) fluxes crash. *Phase I* in the structure of the spring bloom (termed ‘Chl-*a* peak’; Fig. 3) is terminated by a sharp rise in PF and POC fluxes. It is important to note that the POC flux starts to rise first, shortly after February 21st, coeval with an increase in SSTs, while PF fluxes continue to be at minimum at least until February 25th, after which they rise sharply to their annual maximum recorded on March 3rd. Following the initial peak (*phase II*), PF and POC fluxes decline while SSTs stabilize ca. 22 °C. On March 9th, only one week after annual maximum PF flux values are observed, they crash once more until their complete wipeout is recorded on March 19th (*phase III*). In fact, sediment trap samples retrieved between March 19th and April 2nd were practically empty and were therefore not processed for PF picking, assuming they represent an interval of no PF fluxes at all. Yet during the onset of this PF crash, when values start dropping on March 19th, SSTs rise sharply by almost one degree (to ca. 23 °C) simultaneously with a rise in POC values. In both cases, this peak is short lived and after reaching a peak on March 16th they crash as well. Hence, PF and POC values display corresponding minimum values that coincide with a temporal SST drop. All three parameters start rising again after April 1st. Relative to the peak of *phase I*, Chl-*a* values display much more mild fluctuations, though their trends appear to correspond to the above described dynamics. During *phase III*, Chl-*a* values show a minor increase, possibly mimicking the mirror image trend identified during *phase I*. It is important to reiterate that the above-described structure of the 2016 event is largely consistent with the sequence of March–April 2015, indicating the observed patterns are systematic, rather than reflecting sampling biases or random signals within natural variability. In addition, previous comparisons of Chl-*a* surface concentrations between monthly resolved sampling in the deep GOA (*station A*) and daily resolved sampling at the IUI shore, display strong consistency^42^. Thus, the combined evidence suggests a systematic driving mechanism rather than chaotic seawater circulation patterns (e.g., eddies or patchiness^43^) is the fundamental modulator of the observed daily timescale variability of PF fluxes.

Throughout this sequence of events, vertical temperature and fluorescence profiles (Fig. 3) provide additional insight to the driving forces. A drastic temperature drop of ~ 1 °C across the mixed layer during the month leading up to mid-February 2016 (Fig. 3d) does not trigger a notable change in water column fluorescence values (Fig. 3e). However, during the week between February 14th and 21st an additional cooling pulse of the upper ~ 150 m of the water column coincides with an increase in fluorescence values (Fig. 3d, e) and a surface waters Chl-*a* pulse (Fig. 3c), jointly reflecting a phytoplankton bloom that resulted in the development of an intense Chl-*a* maximum ca. 50–100 m (Fig. 3e). This phytoplankton bloom event coincides with a crash in PF fluxes (*phase I*), which we interpret to stem from the retention of thriving PF in the water column resulting in reduced mortality and a drop in the downward vertical flux of foraminifera shells. This is important because it highlights the differences between primary and export production and the fact that the signal of the latter lags that of the former by approximately one week. By February 28th, with the onset of *phase II*, the mixed layer cooling stops and surface waters begin warming, simultaneously with a continued decline in fluorescence values (both absolute and water column-integrated) (Fig. 3f, g), compared with the peak values of PF and POC fluxes (Fig. 3a, b). The surface warming and concurrent drop in integrated fluorescence values (and surface Chl-*a* concentrations) continue into April (Fig. 3h, i).

### Annual flux distribution

Within the annual cycle of PF shell deposition to the sediments, it becomes apparent that a significant fraction of the cumulative annual flux is deposited during several weeks coinciding with the spring phytoplankton bloom. Hence, for the purpose of evaluating the annual cumulative PF flux we focused on the year between April 10, 2015 and April 10, 2016 (Fig. 2a). This choice of time interval, albeit somewhat arbitrary, minimizes biases imposed by the inclusion of both the 2015 and 2016 spring blooms, and while it is clear that inter annual variations should be expected, the designated year provides a good case study for the typical annual patterns familiar from previous studies^29,34,44^. During the first ~ 7 months of the year, between the late spring and the onset of SST cooling in late October, approximately 25% of the total annual content of PF is deposited. Over the subsequent ~ 3.5 months of winter cooling and intermediate PF fluxes, another 45% of the annual content is deposited. The remainder of the PF however, consisting of approximately 32% of the total annual flux, is deposited over a period of 5 weeks in February and March. Moreover, neglecting the intermittent periods during which PF fluxes are negligible (e.g., *phases I and III*), the first event (February 10th to 17th) yields about 9% of the annual flux while the second event (February 25th to March 18th) yields 21% of the annual flux. Considering that about a third of the annual flux of PF is deposited over ~ 4 weeks, this implies any interpretation of the PF content in downcore records is heavily biased toward representing spring and winter conditions in the GOA, with similar implications for other seasonally stratified regions^13,45,46^.

### Planktonic foraminifera species assemblage and succession

The differences in succession timing between the species (Fig. 4) might stem from their food requirements, as PF feeding behavior is species-specific and varies between spinose to non-spinose species. Although spinose species are carnivorous and prefer zooplankton protein over phytoplankton proteins, their juvenile and neanic stages (~ 80 µm shell-size) are mostly phytoplankton grazers^2,47,48^. Non-spinose PF species are omnivorous with preference to herbivorous diet, while diatoms are the major part of their diet^2^. *T. clarkei* (spinose, symbiont-barren), the most dominant species in the northern GOA^38^, is a deep dweller (> 200 m depth habitat^38^) and the smallest PF species with a maximal diameter < 150 µm^49^. *T. quinqueloba* is a spinose, symbiont-barren PF and a relative of *T. clarkei*, although somewhat larger^2,50^. The feeding behavior of both species is largely unknown, though considering the GOA phytoplankton and micro-zooplankton composition, these species are generalists^51^ and might feed on cyanobacteria such as *Synechococcus*, small eukaryotic cells, as well as microzooplankton. Thus, *T. clarkei* and *T. quinqueloba* maximal fluxes correlate with late winter- early spring blooms. *G. ruber* is the most herbivores species (capable of obtaining more energy input from primary producers) among the symbiont bearing spinose species, which makes it less dependent on the consumption of zooplankton^52^. This adaptive feeding behavior enables this species to be more competitive in oligotrophic regions and explains its early peak relative to the other spinose species^38,51^.

Unlike most of the species fluxes that gradually increase during the winter cooling, *G. glutinata*, *G. siphonifera* and *O. universa* fluxes increase and peak almost exclusively during late spring bloom (Fig. 4), reflecting their preference to a well-developed phytoplanktonic community^2^ or to specific zooplanktonic organisms which bloom coevally. Thus, these species define a unique ‘spring species assemblage’, which is of great importance for further interpretation of downcore records at the GOA and elsewhere.

Reproductive cycles of the different PF species are not consistent in their intensity across the annual cycle, reflecting the water column dynamics that impose subsequent changes in food availability. It is possible that PF food scarcity in oligotrophic settings can cause individual PF to skip or delay a reproductive cycle^45^ in order to gain more food, or further grow prior to reproduction as was documented by Spindler et al.^52^, or reproduce out-of-sync when terminal stage is reached. According to the trait-based model^51^, adult PF in oligotrophic environments are impacted by resource competition, resulting in their feeding on a wider range of prey size (than the optimal size). Thus, they are generalist herbivorous or omnivorous (or use other resources as symbiosis) which might alter their life-span according to their feeding life-history.

A long standing paradigm regarding planktonic foraminifera is their lunar-phased synchronized reproduction^16,53,54^, argued to greatly increase chances of successful reproduction by enhancing gamete fusion. Indeed, some of the strongest evidence supporting this hypothesis is derived from early observations in the GOA and Red Sea^28,37,55^. The latter however, as well as more recent studies^16,54,56,57^ who have suggested peak fluxes occur on, or several days after the full moon, were either based on short time windows (typically several months) or on observations at a relatively low temporal resolution (weekly to monthly) and therefore could not provide a complete picture of the lunar synchronized cycles compared to longer term seasonal fluctuations in PF patterns. Here, we find no significant lunar periodicity is displayed by any of the PF species (Fig. 5). Moreover, none of the species displayed an increased number of peaks, or fluxes during the third quarter phase, shortly after the full moon (Supplementary Fig. S2). We consider here the potential time lag between water column processes (e.g., surface phytoplankton blooms) and the sinking flux (e.g., PF and POC fluxes monitored by sediment traps). Inherently, there is not only a time lag between the two time series, as seen in Supplementary Figs. S4–S7, but it is also likely that a peak in reproduction cycles, potentially associated with a lunar signal, could be temporally smeared in the sinking flux, such that its detection (in the sinking flux) is more difficult. When applying stricter criteria (10% of the maximum PF flux), the species *T. quinqueloba* and *D. anfracta* are the only ones to display an increase in peak counts (though not in their flux values) after the full moon (Supplementary Fig. S3). The combined evidence does not support lunar phasing of PF fluxes, or at the least, indicates that the influence of lunar phasing is small, with a negligible impact on absolute flux patterns. It is further noteworthy that observed PF fluxes are low and invariable throughout at least half the year (spring to fall), rendering the potential impact of lunar periodicity to be, from a practical point of view, undetectable for that time interval. It should also be noted that lunar periodicity has been previously associated with fluxes of adult individuals and hence, a more comprehensive evaluation of its impact should be performed on different shell size groups, which we will pursue in a forthcoming publication.

Finally, we note that the somewhat erratic daily timescale variations in PF species fluxes could reflect, at least in part, episodic mortality events resulting from predation and pathogens (diseases) that have yet to be studied or quantified^2^. Another process to consider in this context is asexual reproduction-derived fluxes, which could drive a more rapid population growth than sexual reproduction, resulting in abrupt short-term increases in fluxes^58,59^.

## Conclusions

High resolution daily timescale time series of planktonic foraminifera fluxes, surface Chl-*a* concentrations, and SSTs, supported by corresponding vertical profiles at lower resolution, suggest that seasonal PF flux patterns in the oligotrophic setting of the GOA, northern Red Sea, are controlled by the temperature driven configuration of the water column, with approximately a third of the total annual PF flux delivered over a short period of ~ 4 weeks associated with the spring bloom. Moreover, PF species flux patterns appear to be independent of lunar periodicity, challenging this long-standing paradigm. It is possible that this reflects the smearing of the primary reproductive peak signal in the sinking flux collected in the sediment traps.

On shorter, daily timescales, the dynamics of PF successions are described in detail, particularly during the spring, when the highest annual PF flux lags by a few days the annual surface Chl-*a* peak. Along the phytoplanktonic spring blooms, maximal Chl-*a* surface concentrations are coeval with minimum PF fluxes. The latter peak only about 1–2 weeks later. We interpret this to reflect thriving of succession PF coeval with the Chl-*a* peak, thereby temporarily inhibiting the downward flux of PF shells. Perturbations of PF fluxes during winter months are not related with such prominent Chl-*a* changes, suggesting that at least several PF species (e.g., *G. ruber*, *G. calida*, *D. anfracta*) are modulated by additional food sources. By contrast, *G. glutinata*, *g. siphonifera* and *O. universa*, demonstrate peak fluxes associated almost exclusively with late spring phytoplankton blooms.

Importantly, the highly resolved PF species succession cycles studied here across more than a full annual cycle, reveals that the succession patterns (lifespans) are not consistent throughout the year, nor do the different species necessarily show distinct reproductive patterns.

These findings emphasize the importance of acquiring long term high-resolution time series of biological, physical and geochemical evidence in the oceans, and in particular in remote, under-studied locations or environments such as oligotrophic seas. Moreover, this and comparable studies provide baseline values that are particularly important in light of global climate trends and anthropogenic stressors that are already driving significant changes in PF calcification rates and global distribution patterns^6–9^.

## Methods

### Sampling

An automated time-series sediment trap (McLane PARFLUX-II, aperture area 0.5 m^2^) is deployed continuously since April 2014 at the northern GOA (29° 28.95′ N, 34° 56.22′ E, water depth ~ 605 m) at a depth of ~ 410 m (Supplementary Fig. S1). This site is proximate to the well-studied *station A*, where the vertical temperature, salinity, nutrients and Chl-*a* profiles described here were sampled (see below). The sediment trap is deployed along the same mooring described by Chernihovsky et al.^38^, and a detailed description of the sample processing procedures is given there. In brief, twenty-one bottom cups were filled with a saturated NaCl brine poisoned to ~ 150 mg/l HgCl_2_ in order to minimize sample degradation. The bottom cups were rotated approximately every 1–3 days and mooring retrieval and redeployment was performed approximately every month (Supplementary Table S1). Following sample retrieval, they were transferred within ~ 1–2 h to overnight refrigeration in the lab, allowing suspended particles to settle. Subsequently, each sample was sieved through a 1 mm sieve to remove large organisms, rinsed three times, freeze-dried and weighted for determination of the bulk flux. The bulk samples were then soaked for 3 days in water softener (1% sodium hexametaphosphate buffered with NaOH to pH > 8.2) and then split using a wet sample divider (WSD-10, McLane Research Laboratories) to obtain a subsample of ~ 20 mg. In this study, a sub-set of samples collected between March 2015 and April 2016 was further processed and picked for their PF content and species assemblage. The designated split was soaked in 3% sodium hypochlorite for 8–12 h, rinsed and wet-sieved through a 63 µm sieve. The > 63 µm fraction sample was freeze-dried. Planktonic foraminifera shells were picked from the dried samples and identified to species level using a stereomicroscope (Leica, M205 C). When PF numbers were extremely low, an additional split was processed. Species identification followed Hemleben et al.^47^, Hottinger et al.^60^ and Brummer & Kroon^61^. Broken shells (> 50% shell), containing proloculus and very small (neanic) unidentified stages were classified as "Unidentified" (Supplementary Table S2).

### Particulate organic and inorganic carbon

Inorganic carbon was determined on the bulk sediment by acid titration using an UIC coulometer. Total carbon was determined using a CHN analyzer. Organic carbon was derived by subtracting inorganic carbon from total carbon. The combined analytical error, based on frequent measurements of pure carbonate and certified reference materials is ± 3% SD.

### Oceanographic data

Chl-*a* concentrations and sea surface temperatures (SST) were measured daily by the *Israel National Monitoring Program (NMP)* (https://www.iui-eilat.ac.il/Research/NMPmeteodata.aspx) at the pier of the Interuniversity Institute (IUI) for Marine Sciences at Eilat (Supplementary Fig. S1) and have been shown to be consistent with coeval values at *station A*^42^. Additionally, the NMP measured monthly profiles of water column temperatures, salinity, Chl-*a* concentrations, nutrient contents (TON, NO_2_^−^, NO_3_^−^, PO_4_^3−^, Si(OH)_4_)) at *Station A* (29° 28.22′ N, 34° 55.50′ E). The mixed layer depth (MLD) was calculated using the variable sigma-*t* criterion equivalent to a 0.2 °C temperature change^62^.

### Data evaluation

Spectral analyses of the observed PF flux records were performed based on the Lomb-Scargle power spectral density (PSD) estimate of unevenly spaced time series^63^. The results did not display a statistically significant peak associated with lunar cycles. For comparison, the observations were interpolated at a daily resolution and analyzed again for their spectral distribution, yielding similar results, with the exception of a minor peak of *G. ruber* ca. 30 days. We consider this result and the differences between the two data sets (raw and interpolated) to represent artifacts stemming from the interpolation procedure. These analyses were replicated after seasonal de-trending, again with only minor differences. Our discussion therefore refers to the spectral analyses of the raw data. To further explore this aspect of the results we identified local maxima (peaks) in each of the PF species, which were required to pass a threshold of being higher than their neighboring samples by at least 1% and 10% of the maximum flux of the relevant PF species. The peaks were then categorized according to the lunar phase of their occurrence.

To explore the dynamics and interplay of PF species flux patterns with environmental parameters, we calculated their cross-correlations with SST, Chl-*a*, Bulk and POC fluxes. The correlation analyses were performed using the *crosscorr* function in MATLAB (version R2017b), considering possible lags and leads of up to 20 days in PF species flux relation to environmental parameters.

## Supplementary information

Supplementary file 1.

Supplementary file 2.
