# Supplementary material for: The daily resolved temperature dependence and structure of planktonic foraminifera blooms
Source: Sci Rep. 2020 Oct 15;10:17456. doi: 10.1038/s41598-020-74342-z (PMC7562931; doi:10.1038/s41598-020-74342-z)
Supplement: Supplementary file 1 [file 41598_2020_74342_MOESM1_ESM.docx]

**Supplementary information**

**Figure S1.** Location map. The maps were created using ArcGIS software, version 10.2.1 by Esri ([www.esri.com](http://www.esri.com/)). Global location map was created using MATLAB software, version R2017b ([www.mathworks.com/products/matlab.html](http://www.mathworks.com/products/matlab.html)).

**Figure S2.** Phasing of the lunar cycle in PF shell fluxes. Local maxima (peaks) were identified for each PF species when they passed a threshold of being higher than their neighboring samples by at least 1% of the maximum flux of the specific PF species. The results are sorted according to the quarter phase of their timing. The left ordinate pertains to the PF flux values (# m^-2^ d^-1^) of each peak (black circles) that passed the threshold, and the right ordinate pertains to the count of the number of peaks (*N*) that passed the above threshold (orange bars). The lunar cycle is presented as a dashed grey curve and is divided into 4 quarters, black filled circle along the lower abscissa denotes full moon.

**Figure S3.** Same as Fig. S2, but for a threshold value of 10%.

**Figure S4.** Cross correlations between Total PF, *T. clarkei*, *T. quinqueloba*, and SST, Chl-*a*, bulk and POC fluxes. Each species includes couplets of an upper cross correlation diagram and lower time series plot for comparison. The cross correlation plots examine lags and leads of up to 20 days. Blue horizontal lines mark the upper and lower confidence bounds (2σ).

**Figure S5.** Cross correlations. Same as Fig. S4 but for *G. ruber*, *G. glutinata* and *G. rubescens + G. tenellus*.

**Figure S6.** Cross correlations. Same as Fig. S4 but for *D. anfracta*, *G. siphonifera* and *G. calida.*

**Figure S7.** Cross correlations. Same as Fig. S4 but for *O. universa* and unidentified tests.

**Tables**

**Table S1**. Bulk and Particulate Organic Carbon fluxes. Times pertain to local time.

**Table S2.** Total and species-specific planktonic foraminifera shell fluxes (# m^-2^ d^-1^).

**Table S3.** Correlation coefficients of planktonic foraminifera species flux, moon illumination, SST, Chl-*a*, particulate bulk and POC fluxes.
