# Supplementary material for: The daily resolved temperature dependence and structure of planktonic foraminifera blooms
Source: Sci Rep. 2020 Oct 15;10:17456. doi: 10.1038/s41598-020-74342-z (PMC7562931; doi:10.1038/s41598-020-74342-z)
Supplement: Supplementary file 2 [file 41598_2020_74342_MOESM2_ESM.pdf]

Supplementary material

The daily resolved temperature dependence and structure of planktonic foraminifera blooms

Chernihovsky N.[1,2*], Almogi-Labin A.[3], Kienast S.S. [4], Torfstein A.[1,2]

[1] The Fredy & Nadine Herrmann Institute of Earth Sciences, The Hebrew University of Jerusalem, Jerusalem 9190401, Israel
[2] The Interuniversity Institute for Marine Sciences, Eilat 8810302, Israel
[3] Geological Survey of Israel, 32 Yesha'ayahu Leibowitz Street, Jerusalem 9692100, Israel
[4] Department of Oceanography, Dalhousie University, Halifax, Nova Scotia, B3H 4R2, Canada

*Correspondence to natalie.chernihovsk@mail.huji.ac.il

Figure S1

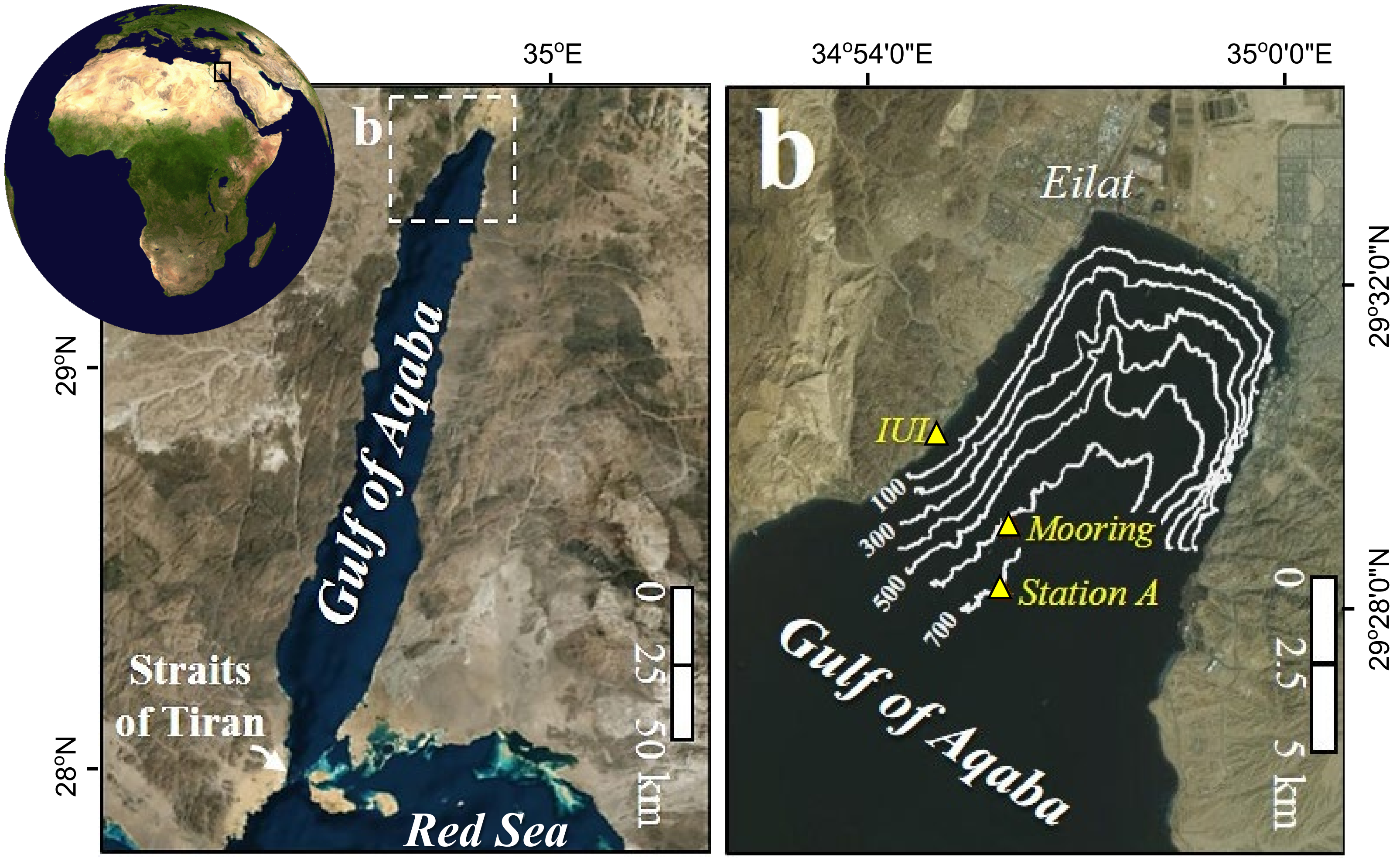

35°E
b
Gulf of Aqaba
Straits of Tiran
Red Sea
29°N
28°N
0 25 50 km
34°54'0"E
35°0'0"E
29°32'0"N
29°28'0"N
b
Eilat
IUI
100
300
500
700
Mooring
Station A
Gulf of Aqaba
0 2.5 5 km

Figure S2

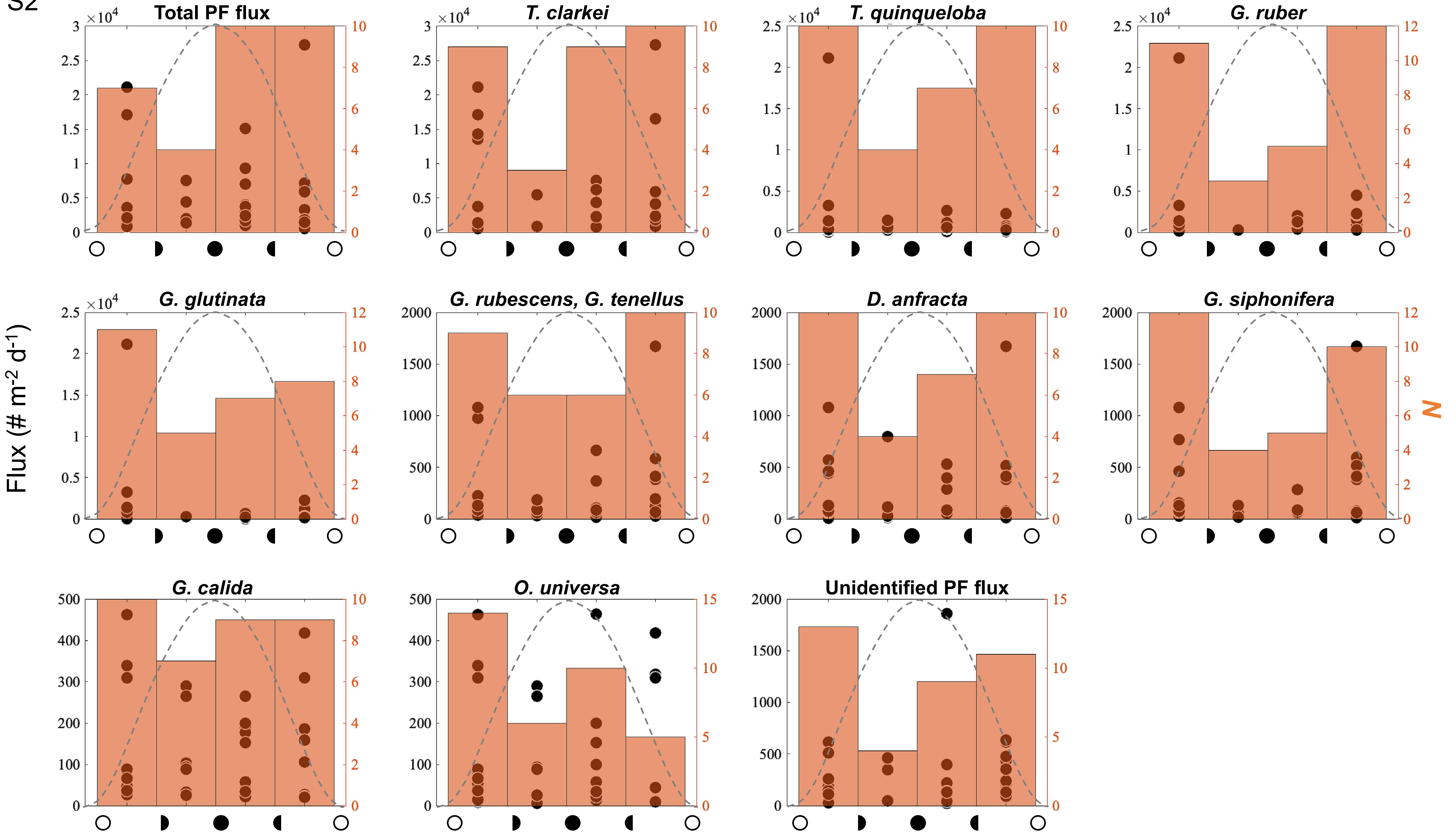

Total PF flux
T. clarkei
T. quinqueloba
G. ruber
G. glutinata
G. rubescens, G. tenellus
D. anfracta
G. siphonifera
G. calida
O. universa
Unidentified PF flux
Flux (# m-2 d-1)
N

Figure S3

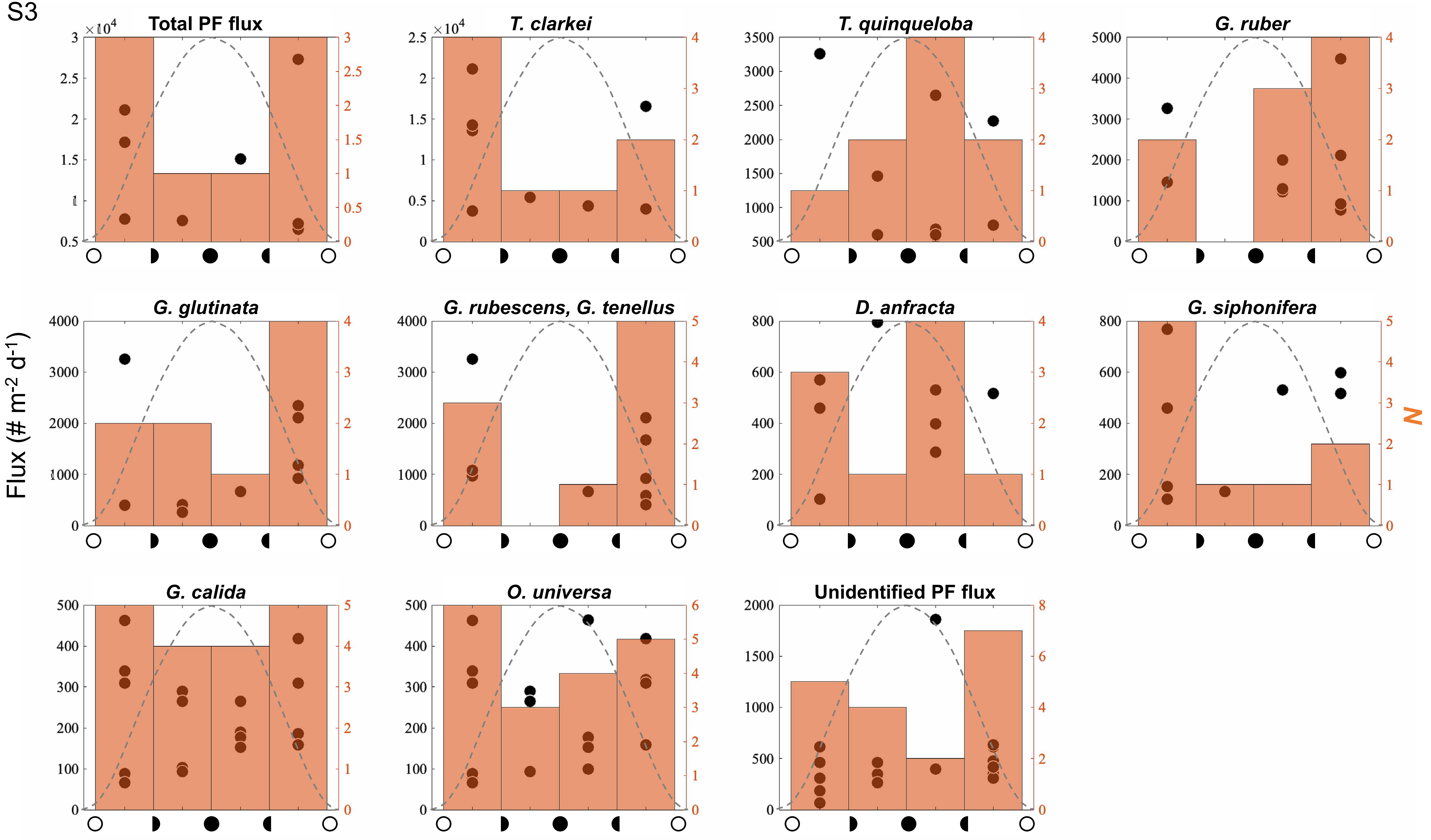

Total PF flux
T. clarkei
T. quinqueloba
G. ruber
G. glutinata
G. rubescens, G. tenellus
D. anfracta
G. siphonifera
G. calida
O. universa
Unidentified PF flux
Flux (# m-2 d-1)
N

Figure S4

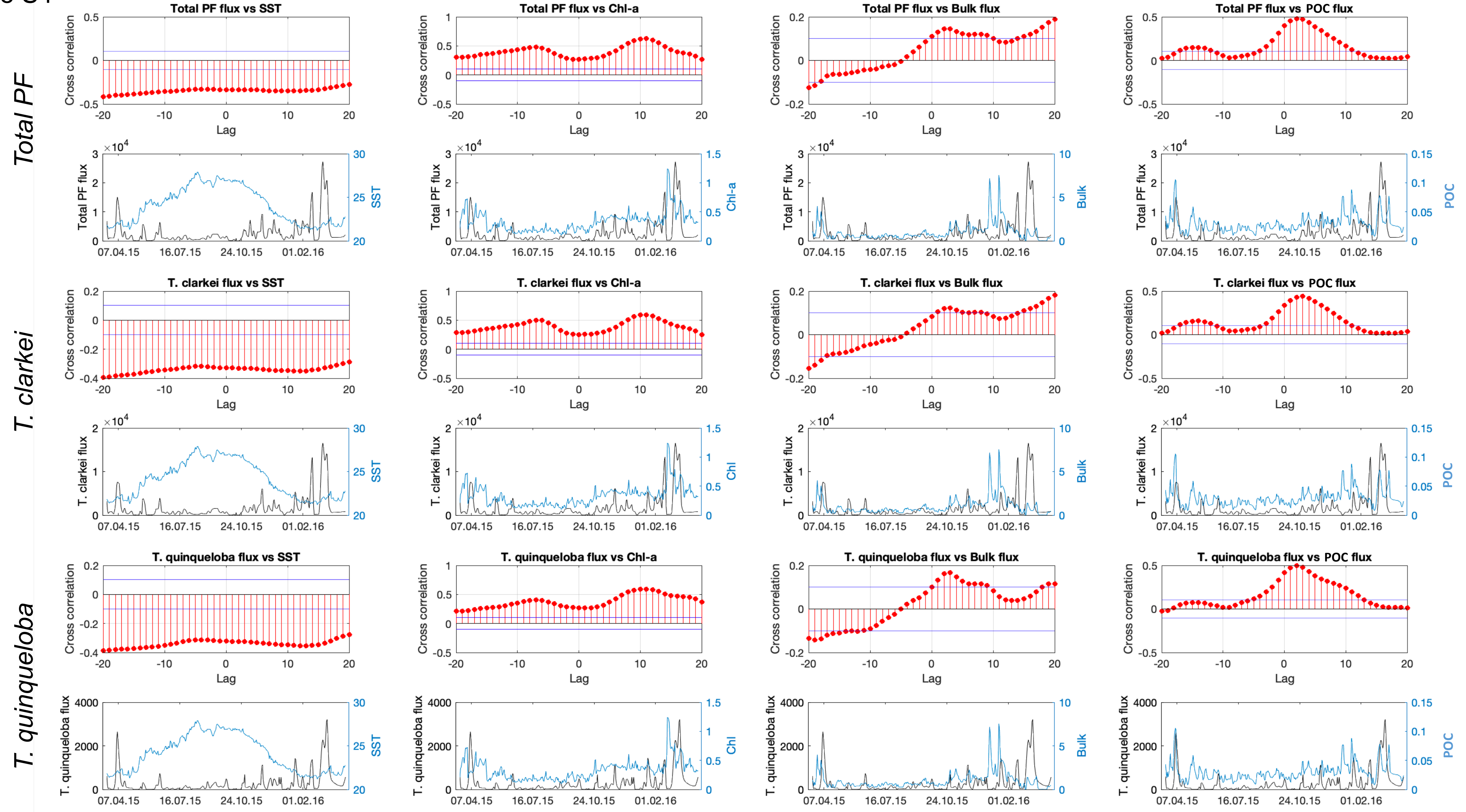
Total PF
T. clarkei
T. quinqueloba
Total PF flux vs SST
Total PF flux vs Chl-a
Total PF flux vs Bulk flux
Total PF flux vs POC flux
T. clarkei flux vs SST
T. clarkei flux vs Chl-a
T. clarkei flux vs Bulk flux
T. clarkei flux vs POC flux
T. quinqueloba flux vs SST
T. quinqueloba flux vs Chl-a
T. quinqueloba flux vs Bulk flux
T. quinqueloba flux vs POC flux
Cross correlation
Lag
Total PF flux
T. clarkei flux
T. quinqueloba flux
SST
Chl-a
Chl
Bulk
POC
07.04.15
16.07.15
24.10.15
01.02.16

Figure S5

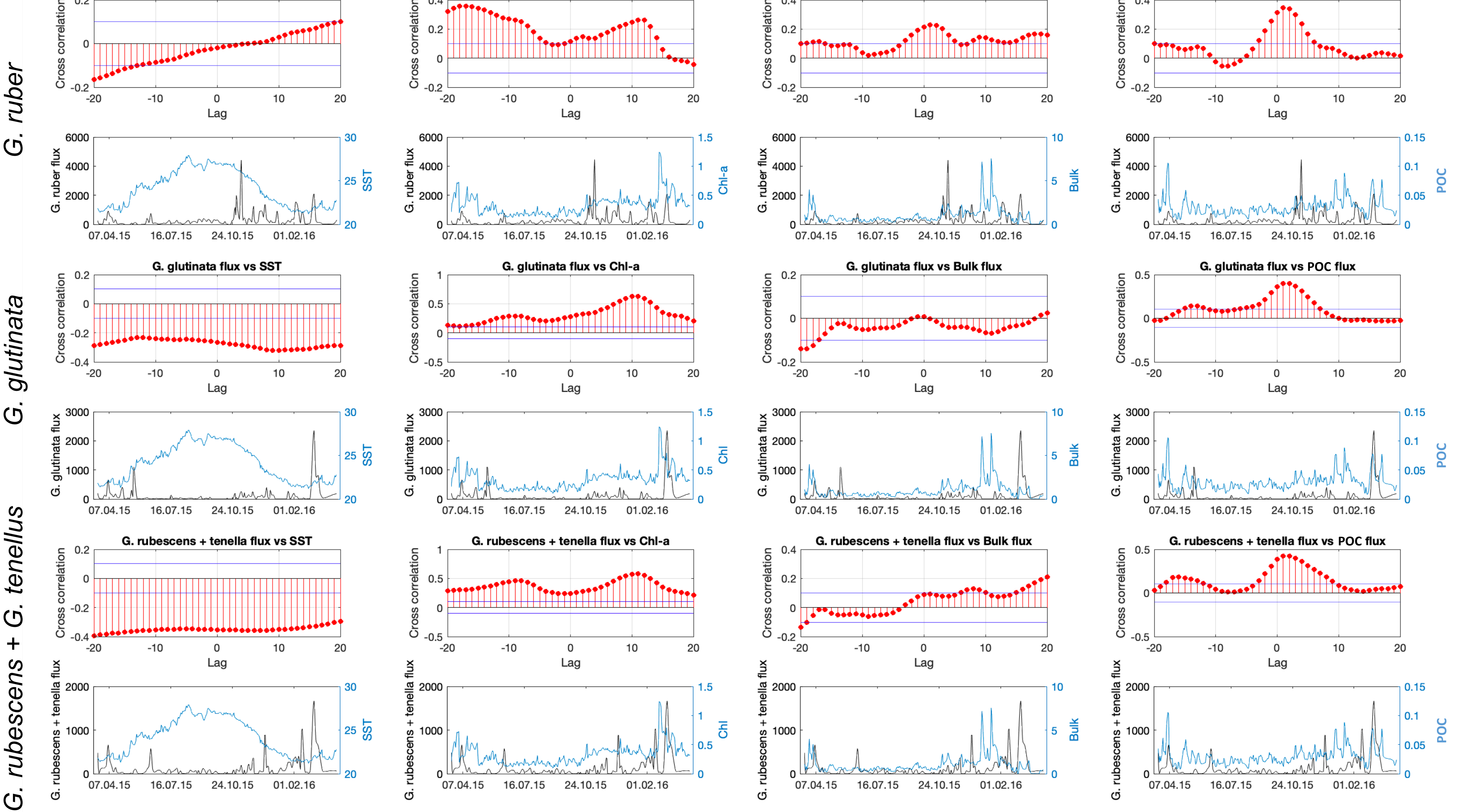
G. ruber
G. glutinata
G. rubescens + G. tenellus
G. ruber flux vs SST
G. ruber flux vs Chl-a
G. ruber flux vs Bulk flux
G. ruber flux vs POC flux
G. glutinata flux vs SST
G. glutinata flux vs Chl-a
G. glutinata flux vs Bulk flux
G. glutinata flux vs POC flux
G. rubescens + tenella flux vs SST
G. rubescens + tenella flux vs Chl-a
G. rubescens + tenella flux vs Bulk flux
G. rubescens + tenella flux vs POC flux
Cross correlation
Lag
G. ruber flux
G. glutinata flux
G. rubescens + tenella flux
SST
Chl-a
Chl
Bulk
POC
07.04.15
16.07.15
24.10.15
01.02.16

Figure S6

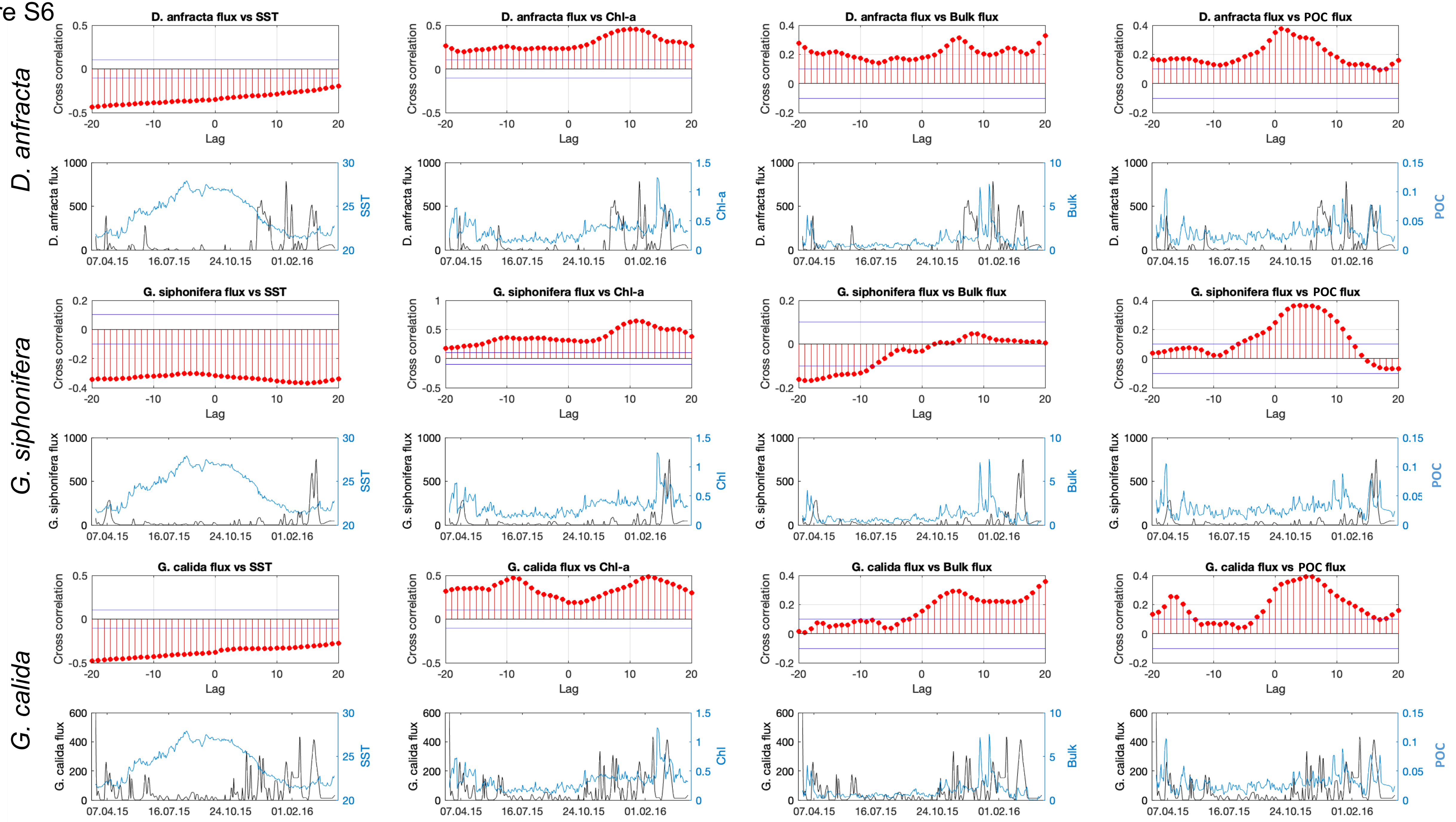
D. anfracta
G. siphonifera
G. calida
D. anfracta flux vs SST
D. anfracta flux vs Chl-a
D. anfracta flux vs Bulk flux
D. anfracta flux vs POC flux
G. siphonifera flux vs SST
G. siphonifera flux vs Chl-a
G. siphonifera flux vs Bulk flux
G. siphonifera flux vs POC flux
G. calida flux vs SST
G. calida flux vs Chl-a
G. calida flux vs Bulk flux
G. calida flux vs POC flux
Cross correlation
Lag
D. anfracta flux
G. siphonifera flux
G. calida flux
SST
Chl-a
Chl
Bulk
POC
07.04.15
16.07.15
24.10.15
01.02.16

Figure S7

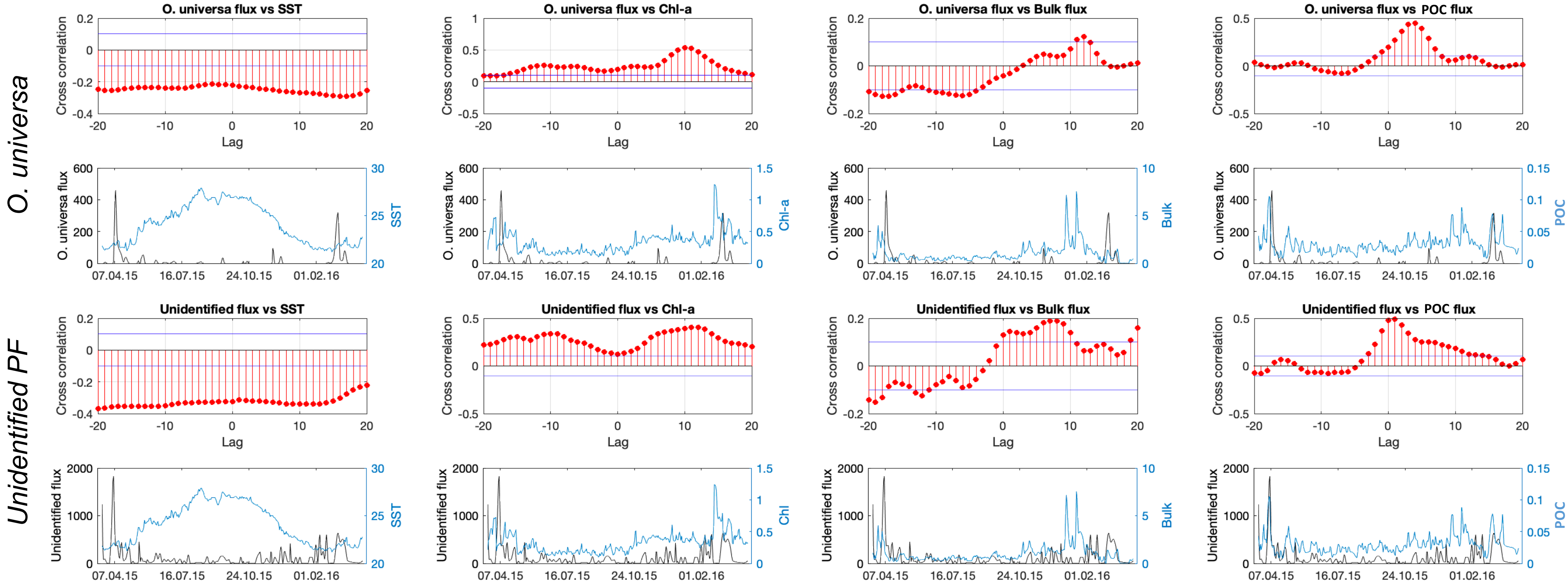

O. universa
Unidentified PF
O. universa flux vs SST
O. universa flux vs Chl-a
O. universa flux vs Bulk flux
O. universa flux vs POC flux
Unidentified flux vs SST
Unidentified flux vs Chl-a
Unidentified flux vs Bulk flux
Unidentified flux vs POC flux
Cross correlation
Lag
O. universa flux
Unidentified flux
SST
Chl-a
Chl
Bulk
POC
07.04.15
16.07.15
24.10.15
01.02.16

Table S1. Bulk and Particulate Organic Carbon fluxes. Times pertain to local time.

Sample	Start time	End time	Interval (hours)	Water depth (m)	Trap depth (m)	Bulk (g d^{-1} m^{-2})	POC (g C d^{-1} m^{-2})
1	17.03.2015 18:00	18.03.2015 18:00	24	604	405	1.11	0.04
2	18.03.2015 18:00	19.03.2015 18:00	24	604	405	0.85	0.03
3	19.03.2015 18:00	20.03.2015 06:00	12	604	405	0.54	0.02
4	20.03.2015 06:00	20.03.2015 18:00	12	604	405	0.36	0.02
5	20.03.2015 18:00	21.03.2015 06:00	12	604	405	0.56	0.02
6	21.03.2015 06:00	21.03.2015 18:00	12	604	405	1.04	0.04
7	21.03.2015 18:00	22.03.2015 06:00	12	604	405	0.50	0.02
8	23.03.2015 18:00	25.03.2015 06:00	36	603	405	1.48	0.03
9	25.03.2015 06:00	26.03.2015 18:00	36	603	405	4.12	0.06
10	26.03.2015 18:00	28.03.2015 06:00	36	603	405	2.25	0.04
11	28.03.2015 06:00	29.03.2015 18:00	36	603	405	2.27	0.04
12	29.03.2015 18:00	31.03.2015 06:00	36	603	405	1.54	0.03
13	31.03.2015 06:00	01.04.2015 18:00	36	603	405	3.44	0.08
14	01.04.2015 18:00	03.04.2015 06:00	36	603	405	2.44	0.10
15	03.04.2015 06:00	04.04.2015 18:00	36	603	405	2.38	0.11
16	04.04.2015 18:00	06.04.2015 06:00	36	603	405	1.08	0.04
17	06.04.2015 06:00	07.04.2015 18:00	36	603	405	0.89	0.03
18	07.04.2015 18:00	09.04.2015 06:00	36	603	405	0.69	0.02
19	09.04.2015 06:00	10.04.2015 18:00	36	603	405	0.64	0.03
20	10.04.2015 18:00	12.04.2015 06:00	36	603	405	0.22	0.01
21	12.04.2015 06:00	13.04.2015 18:00	36	603	405	0.28	0.01
22	13.04.2015 18:00	15.04.2015 06:00	36	603	405	0.62	0.02
23	15.04.2015 06:00	16.04.2015 18:00	36	603	405	0.56	0.02
24	16.04.2015 18:00	18.04.2015 06:00	36	603	405	0.26	0.01
25	18.04.2015 06:00	19.04.2015 18:00	36	603	405	0.19	0.01
26	19.04.2015 18:00	21.04.2015 06:00	36	603	405	0.65	0.02
27	21.04.2015 06:00	22.04.2015 18:00	36	603	405	0.22	0.01
28	22.04.2015 18:00	24.04.2015 06:00	36	603	405	0.23	0.01
29	26.04.2015 18:00	28.04.2015 06:00	36	603	398	0.91	0.03
30	28.04.2015 06:00	29.04.2015 18:00	36	603	398	1.07	0.04
31	29.04.2015 18:00	01.05.2015 06:00	36	603	398	0.89	0.04
32	01.05.2015 06:00	02.05.2015 18:00	36	603	398	1.01	0.06
33	02.05.2015 18:00	04.05.2015 06:00	36	603	398	1.03	0.05
34	04.05.2015 06:00	05.05.2015 18:00	36	603	398	0.70	0.03
35	05.05.2015 18:00	07.05.2015 06:00	36	603	398	0.62	0.03
36	07.05.2015 06:00	08.05.2015 18:00	36	603	398	0.42	0.02
37	08.05.2015 18:00	10.05.2015 06:00	36	603	398	0.38	0.02
38	10.05.2015 06:00	11.05.2015 18:00	36	603	398	0.46	0.02
39	11.05.2015 18:00	13.05.2015 06:00	36	603	398	0.69	0.04
40	13.05.2015 06:00	14.05.2015 18:00	36	603	398	0.70	0.04
41	14.05.2015 18:00	16.05.2015 06:00	36	603	398	0.67	0.03
42	16.05.2015 06:00	17.05.2015 18:00	36	603	398	0.73	0.03
43	17.05.2015 18:00	19.05.2015 06:00	36	603	398	0.44	0.02
44	19.05.2015 06:00	20.05.2015 18:00	36	603	398	0.45	0.02
45	20.05.2015 18:00	22.05.2015 06:00	36	603	398	0.62	0.03
46	22.05.2015 06:00	23.05.2015 18:00	36	603	398	0.69	0.04
47	23.05.2015 18:00	25.05.2015 06:00	36	603	398	0.47	0.03
48	25.05.2015 06:00	26.05.2015 18:00	36	603	398	0.32	
49	26.05.2015 18:00	28.05.2015 06:00	36	603	398	0.36	0.02
50	02.06.2015 18:00	04.06.2015 06:00	36	606	415	0.38	0.02
51	04.06.2015 06:00	05.06.2015 18:00	36	606	415	0.68	0.04
52	05.06.2015 18:00	07.06.2015 06:00	36	606	415	0.65	0.03
53	07.06.2015 06:00	08.06.2015 18:00	36	606	415	0.60	0.03
54	08.06.2015 18:00	10.06.2015 06:00	36	606	415	0.63	0.03
55	10.06.2015 06:00	11.06.2015 18:00	36	606	415	0.63	0.03
56	11.06.2015 18:00	13.06.2015 06:00	36	606	415	0.77	0.03
57	13.06.2015 06:00	14.06.2015 18:00	36	606	415	0.54	0.02
58	14.06.2015 18:00	16.06.2015 06:00	36	606	415	0.35	0.02
59	16.06.2015 06:00	17.06.2015 18:00	36	606	415	0.63	0.02
60	17.06.2015 18:00	19.06.2015 06:00	36	606	415	0.25	

Table S1. Cont.

Sample	Start time	End time	Interval (hours)	Water depth (m)	Trap depth (m)	Bulk (g d^{-1} m^{-2})	POC (g C d^{-1} m^{-2})
61	19.06.2015 06:00	20.06.2015 18:00	36	606	415	0.33	0.01
62	20.06.2015 18:00	22.06.2015 06:00	36	606	415	0.34	0.01
63	22.06.2015 06:00	23.06.2015 18:00	36	606	415	0.58	0.03
64	23.06.2015 18:00	25.06.2015 06:00	36	606	415	0.38	0.02
65	25.06.2015 06:00	26.06.2015 18:00	36	606	415	0.33	0.02
66	26.06.2015 18:00	28.06.2015 06:00	36	606	415	0.22	
67	28.06.2015 06:00	29.06.2015 18:00	36	606	415	0.33	0.02
68	29.06.2015 18:00	01.07.2015 06:00	36	606	415	0.25	
69	01.07.2015 06:00	02.07.2015 18:00	36	606	415	0.38	0.02
70	02.07.2015 18:00	04.07.2015 06:00	36	606	415	0.61	0.03
71	05.07.2015 18:00	07.07.2015 06:00	36	608	408	0.31	0.01
72	07.07.2015 06:00	08.07.2015 18:00	36	608	408	0.45	0.01
73	08.07.2015 18:00	10.07.2015 06:00	36	608	408	0.73	
74	10.07.2015 06:00	11.07.2015 18:00	36	608	408	0.45	0.01
75	11.07.2015 18:00	13.07.2015 06:00	36	608	408	0.58	0.02
76	13.07.2015 06:00	14.07.2015 18:00	36	608	408	0.80	
77	14.07.2015 18:00	16.07.2015 06:00	36	608	408	0.86	0.03
78	16.07.2015 06:00	17.07.2015 18:00	36	608	408	0.75	0.03
79	17.07.2015 18:00	19.07.2015 06:00	36	608	408	0.49	0.02
80	19.07.2015 06:00	20.07.2015 18:00	36	608	408	0.51	0.02
81	20.07.2015 18:00	22.07.2015 06:00	36	608	408	0.67	0.03
82	22.07.2015 06:00	23.07.2015 18:00	36	608	408	0.64	
83	23.07.2015 18:00	25.07.2015 06:00	36	608	408	0.66	0.02
84	25.07.2015 06:00	26.07.2015 18:00	36	608	408	0.64	0.03
85	26.07.2015 18:00	28.07.2015 06:00	36	608	408	0.40	0.02
86	28.07.2015 06:00	29.07.2015 18:00	36	608	408	0.53	0.02
87	29.07.2015 18:00	31.07.2015 06:00	36	608	408	0.63	0.03
88	31.07.2015 06:00	01.08.2015 18:00	36	608	408	0.66	
89	01.08.2015 18:00	03.08.2015 06:00	36	608	408	0.32	0.02
90	03.08.2015 06:00	04.08.2015 18:00	36	608	408	0.60	
91	04.08.2015 18:00	06.08.2015 06:00	36	608	408	0.27	0.02
92	11.08.2015 18:00	13.08.2015 06:00	36	604	412	0.26	0.01
93	13.08.2015 06:00	14.08.2015 18:00	36	604	412	0.59	0.03
94	14.08.2015 18:00	16.08.2015 06:00	36	604	412	0.70	0.03
95	16.08.2015 06:00	17.08.2015 18:00	36	604	412	0.79	0.03
96	17.08.2015 18:00	19.08.2015 06:00	36	604	412	0.71	0.02
97	19.08.2015 06:00	20.08.2015 18:00	36	604	412	0.87	0.03
98	20.08.2015 18:00	22.08.2015 06:00	36	604	412	0.66	0.02
99	22.08.2015 06:00	23.08.2015 18:00	36	604	412	0.62	0.02
100	23.08.2015 18:00	25.08.2015 06:00	36	604	412	0.65	0.03
101	25.08.2015 06:00	26.08.2015 06:00	24	604	412	0.69	0.02
102	26.08.2015 06:00	27.08.2015 06:00	24	604	412	0.43	0.01
103	27.08.2015 06:00	28.08.2015 06:00	24	604	412	0.50	0.02
104	28.08.2015 06:00	29.08.2015 06:00	24	604	412	0.70	0.02
105	29.08.2015 06:00	30.08.2015 06:00	24	604	412	0.44	0.03
106	30.08.2015 06:00	31.08.2015 06:00	24	604	412	0.67	0.03
107	31.08.2015 06:00	01.09.2015 06:00	24	604	412	0.63	0.03
108	01.09.2015 06:00	02.09.2015 06:00	24	604	412	0.65	0.03
109	02.09.2015 06:00	03.09.2015 06:00	24	604	412	0.65	0.03
110	03.09.2015 06:00	04.09.2015 06:00	24	604	412	0.49	0.03
111	04.09.2015 06:00	05.09.2015 06:00	24	604	412	0.67	0.02
112	05.09.2015 06:00	06.09.2015 06:00	24	604	412	0.59	0.02
113	07.09.2015 18:00	09.09.2015 02:00	32	608	408	0.49	0.02
114	09.09.2015 02:00	10.09.2015 10:00	32	608	408	0.60	0.02
115	10.09.2015 10:00	11.09.2015 18:00	32	608	408	0.82	0.02
116	11.09.2015 18:00	13.09.2015 02:00	32	608	408	0.95	0.03
117	13.09.2015 02:00	14.09.2015 10:00	32	608	408	1.04	0.03
118	14.09.2015 10:00	15.09.2015 18:00	32	608	408	1.22	0.04
119	15.09.2015 18:00	17.09.2015 02:00	32	608	408	1.00	0.03
120	17.09.2015 02:00	18.09.2015 10:00	32	608	408	1.39	0.03

Table S1. Cont.

Sample	Start time	End time	Interval (hours)	Water depth (m)	Trap depth (m)	Bulk (g d^{-1} m^{-2})	POC (g C d^{-1} m^{-2})
121	18.09.2015 10:00	19.09.2015 18:00	32	608	408	1.01	0.03
122	19.09.2015 18:00	21.09.2015 02:00	32	608	408	0.62	0.02
123	21.09.2015 02:00	22.09.2015 10:00	32	608	408	0.91	0.03
124	22.09.2015 10:00	23.09.2015 18:00	32	608	408	0.75	0.03
125	23.09.2015 18:00	25.09.2015 02:00	32	608	408	0.66	0.02
126	25.09.2015 02:00	26.09.2015 10:00	32	608	408	0.60	0.03
127	26.09.2015 10:00	27.09.2015 18:00	32	608	408	0.77	0.02
128	27.09.2015 18:00	29.09.2015 02:00	32	608	408	0.64	0.02
129	29.09.2015 02:00	30.09.2015 10:00	32	608	408	0.82	0.02
130	30.09.2015 10:00	01.10.2015 18:00	32	608	408	0.79	0.02
131	01.10.2015 18:00	03.10.2015 02:00	32	608	408	0.51	0.02
132	03.10.2015 02:00	04.10.2015 10:00	32	608	408	0.44	0.02
133	04.10.2015 10:00	05.10.2015 18:00	32	608	408	0.51	0.02
134	07.10.2015 18:00	08.10.2015 22:00	28	600	411	0.32	0.01
135	08.10.2015 22:00	10.10.2015 02:00	28	600	411	0.64	0.03
136	10.10.2015 02:00	11.10.2015 06:00	28	600	411	0.72	0.03
137	11.10.2015 06:00	12.10.2015 10:00	28	600	411	0.48	0.03
138	12.10.2015 10:00	13.10.2015 14:00	28	600	411	0.53	0.02
139	13.10.2015 14:00	14.10.2015 18:00	28	600	411	0.49	0.02
140	14.10.2015 18:00	15.10.2015 22:00	28	600	411	0.58	0.02
141	15.10.2015 22:00	17.10.2015 02:00	28	600	411	0.51	0.02
142	17.10.2015 02:00	18.10.2015 06:00	28	600	411	0.46	0.02
143	18.10.2015 06:00	19.10.2015 10:00	28	600	411	0.43	0.02
144	19.10.2015 10:00	20.10.2015 14:00	28	600	411	0.46	0.02
145	20.10.2015 14:00	21.10.2015 18:00	28	600	411	0.74	0.03
146	21.10.2015 18:00	22.10.2015 22:00	28	600	411	0.89	0.03
147	22.10.2015 22:00	24.10.2015 02:00	28	600	411	0.66	0.03
148	24.10.2015 02:00	25.10.2015 06:00	28	600	411	0.66	0.02
149	25.10.2015 06:00	26.10.2015 10:00	28	600	411	0.90	0.03
150	26.10.2015 10:00	27.10.2015 14:00	28	600	411	1.72	0.05
151	27.10.2015 14:00	28.10.2015 18:00	28	600	411	2.28	0.05
152	28.10.2015 18:00	29.10.2015 22:00	28	600	411	1.38	0.03
153	29.10.2015 22:00	31.10.2015 02:00	28	600	411	0.99	0.03
154	30.10.2015 02:00	31.10.2015 06:00	28	600	411	1.10	0.03
155	02.11.2015 18:00	04.11.2015 02:00	32	605	403	1.46	0.04
156	04.11.2015 02:00	05.11.2015 10:00	32	605	403	1.71	0.04
157	05.11.2015 10:00	06.11.2015 18:00	32	605	403	2.42	0.05
158	06.11.2015 18:00	08.11.2015 02:00	32	605	403	2.11	0.04
159	08.11.2015 02:00	09.11.2015 10:00	32	605	403	1.63	0.04
160	09.11.2015 10:00	10.11.2015 18:00	32	605	403	1.60	0.04
161	10.11.2015 18:00	12.11.2015 02:00	32	605	403	1.12	0.03
162	12.11.2015 02:00	13.11.2015 10:00	32	605	403	1.21	0.02
163	13.11.2015 10:00	14.11.2015 18:00	32	605	403	0.91	0.03
164	14.11.2015 18:00	16.11.2015 02:00	32	605	403	0.86	0.02
165	16.11.2015 02:00	17.11.2015 10:00	32	605	403	0.84	0.02
166	17.11.2015 10:00	18.11.2015 18:00	32	605	403	1.08	0.03
167	18.11.2015 18:00	20.11.2015 02:00	32	605	403	1.17	0.04
168	20.11.2015 02:00	21.11.2015 10:00	32	605	403	1.12	0.03
169	21.11.2015 10:00	22.11.2015 18:00	32	605	403	0.98	0.03
170	22.11.2015 18:00	24.11.2015 02:00	32	605	403	1.14	0.03
171	24.11.2015 02:00	25.11.2015 10:00	32	605	403	1.71	0.04
172	25.11.2015 10:00	26.11.2015 18:00	32	605	403	1.74	0.03
173	26.11.2015 18:00	28.11.2015 02:00	32	605	403	1.94	0.04
174	28.11.2015 02:00	29.11.2015 10:00	32	605	403	2.38	0.05
175	29.11.2015 10:00	30.11.2015 18:00	32	605	403	0.77	0.02
176	02.12.2015 18:00	04.12.2015 00:00	30	613	408	1.41	0.04
177	04.12.2015 00:00	05.12.2015 06:00	30	613	408	1.73	0.03
178	05.12.2015 06:00	06.12.2015 12:00	30	613	408	1.17	0.03
179	06.12.2015 12:00	07.12.2015 18:00	30	613	408	1.57	0.04
180	07.12.2015 18:00	09.12.2015 00:00	30	613	408	1.56	0.04

Table S1. Cont.

Sample	Start time	End time	Interval (hours)	Water depth (m)	Trap depth (m)	Bulk (g d^{-1} m^{-2})	POC (g C d^{-1} m^{-2})
181	09.12.2015 00:00	10.12.2015 06:00	30	613	408	1.42	0.03
182	10.12.2015 06:00	11.12.2015 12:00	30	613	408	1.37	0.03
183	11.12.2015 12:00	12.12.2015 18:00	30	613	408	1.19	0.03
184	12.12.2015 18:00	14.12.2015 00:00	30	613	408	1.15	0.04
185	14.12.2015 00:00	15.12.2015 06:00	30	613	408	1.25	0.03
186	15.12.2015 06:00	16.12.2015 12:00	30	613	408	1.24	0.04
187	16.12.2015 12:00	17.12.2015 18:00	30	613	408	1.38	0.03
188	17.12.2015 18:00	19.12.2015 00:00	30	613	408	1.19	0.02
189	19.12.2015 00:00	20.12.2015 06:00	30	613	408	0.96	0.03
190	20.12.2015 06:00	21.12.2015 12:00	30	613	408	1.04	0.02
191	21.12.2015 12:00	22.12.2015 18:00	30	613	408	1.01	0.03
192	22.12.2015 18:00	24.12.2015 00:00	30	613	408	1.22	0.03
193	24.12.2015 00:00	25.12.2015 06:00	30	613	408	1.41	0.04
194	25.12.2015 06:00	26.12.2015 12:00	30	613	408	1.35	0.03
195	26.12.2015 12:00	27.12.2015 18:00	30	613	408	1.49	0.03
196	27.12.2015 18:00	29.12.2015 00:00	30	613	408	1.42	0.03
197	30.12.2015 18:00	01.01.2016 06:00	36	612	417	7.23	0.08
198	01.01.2016 06:00	02.01.2016 18:00	36	612	417	5.09	0.08
199	02.01.2016 18:00	04.01.2016 06:00	36	612	417	3.15	0.05
200	04.01.2016 06:00	05.01.2016 18:00	36	612	417	2.10	0.04
201	05.01.2016 18:00	07.01.2016 06:00	36	612	417	1.70	0.04
202	07.01.2016 06:00	08.01.2016 18:00	36	612	417	1.84	0.04
203	08.01.2016 18:00	10.01.2016 06:00	36	612	417	3.00	0.04
204	10.01.2016 06:00	11.01.2016 18:00	36	612	417	1.66	0.03
205	11.01.2016 18:00	13.01.2016 06:00	36	612	417	1.46	0.03
206	13.01.2016 06:00	14.01.2016 18:00	36	612	417	2.53	0.04
207	14.01.2016 18:00	16.01.2016 06:00	36	612	417	7.87	0.09
208	16.01.2016 06:00	17.01.2016 18:00	36	612	417	5.23	0.07
209	17.01.2016 18:00	19.01.2016 06:00	36	612	417	3.06	0.05
210	19.01.2016 06:00	20.01.2016 18:00	36	612	417	3.36	0.06
211	20.01.2016 18:00	22.01.2016 06:00	36	612	417	3.30	0.06
212	22.01.2016 06:00	23.01.2016 18:00	36	612	417	3.08	0.05
213	23.01.2016 18:00	25.01.2016 06:00	36	612	417	2.80	0.05
214	25.01.2016 06:00	26.01.2016 18:00	36	612	417		
215	26.01.2016 18:00	28.01.2016 06:00	36	612	417	1.24	0.03
216	28.01.2016 06:00	29.01.2016 18:00	36	612	417	0.79	
217	29.01.2016 18:00	31.01.2016 06:00	36	612	417	2.12	0.04
218	01.02.2016 18:00	03.02.2016 01:00	31	600	403	1.42	0.03
219	03.02.2016 01:00	04.02.2016 08:00	31	600	403	1.55	0.04
220	04.02.2016 08:00	05.02.2016 15:00	31	600	403	1.57	0.03
221	05.02.2016 15:00	06.02.2016 22:00	31	600	403	1.44	0.03
222	06.02.2016 22:00	08.02.2016 05:00	31	600	403	1.24	0.03
223	08.02.2016 05:00	09.02.2016 12:00	31	600	403	0.98	0.03
224	09.02.2016 12:00	10.02.2016 19:00	31	600	403	1.02	0.02
225	10.02.2016 19:00	12.02.2016 02:00	31	600	403	0.87	0.02
226	12.02.2016 02:00	13.02.2016 09:00	31	600	403	1.37	0.03
227	13.02.2016 09:00	14.02.2016 16:00	31	600	403	1.09	0.03
228	14.02.2016 16:00	15.02.2016 23:00	31	600	403	1.09	0.03
229	15.02.2016 23:00	17.02.2016 06:00	31	600	403	0.67	0.02
230	17.02.2016 06:00	18.02.2016 13:00	31	600	403	0.28	0.01
231	18.02.2016 13:00	19.02.2016 20:00	31	600	403	0.74	0.02
232	19.02.2016 20:00	21.02.2016 03:00	31	600	403	0.28	0.01
233	21.02.2016 03:00	22.02.2016 10:00	31	600	403	0.20	0.01
234	22.02.2016 10:00	23.02.2016 17:00	31	600	403	0.26	0.01
235	23.02.2016 17:00	25.02.2016 00:00	31	600	403	0.40	0.02
236	25.02.2016 00:00	26.02.2016 07:00	31	600	403	0.82	0.04
237	26.02.2016 07:00	27.02.2016 14:00	31	600	403	0.04	
238	27.02.2016 14:00	28.02.2016 21:00	31	600	403	0.00	
239	29.02.2016 18:00	02.03.2016 10:00	40	605	417	1.47	0.08
240	02.03.2016 10:00	04.03.2016 02:00	40	605	417	1.17	0.06

Table S1. Cont.

Sample	Start time	End time	Interval (hours)	Water depth (m)	Trap depth (m)	Bulk (g d^{-1} m^{-2})	POC (g C d^{-1} m^{-2})
241	04.03.2016 02:00	05.03.2016 18:00	40	605	417	0.85	0.04
242	05.03.2016 18:00	07.03.2016 10:00	40	605	417	0.58	0.03
243	07.03.2016 10:00	09.03.2016 02:00	40	605	417	0.53	0.03
244	09.03.2016 02:00	10.03.2016 18:00	40	605	417	0.51	0.03
245	10.03.2016 18:00	12.03.2016 10:00	40	605	417	0.53	0.03
246	12.03.2016 10:00	14.03.2016 02:00	40	605	417	1.13	0.05
247	14.03.2016 02:00	15.03.2016 18:00	40	605	417	1.17	0.05
248	15.03.2016 18:00	17.03.2016 10:00	40	605	417	1.54	0.08
249	17.03.2016 10:00	19.03.2016 02:00	40	605	417	0.44	0.03
250	19.03.2016 02:00	20.03.2016 18:00	40	605	417	0.01	
251	20.03.2016 18:00	22.03.2016 10:00	40	605	417	0.00	
252	22.03.2016 10:00	24.03.2016 02:00	40	605	417	0.00	
253	24.03.2016 02:00	25.03.2016 18:00	40	605	417	0.01	
254	25.03.2016 18:00	27.03.2016 10:00	40	605	417	0.01	
255	27.03.2016 10:00	29.03.2016 02:00	40	605	417	0.00	
256	29.03.2016 02:00	30.03.2016 18:00	40	605	417	0.00	
257	30.03.2016 18:00	01.04.2016 10:00	40	605	417	0.00	
258	01.04.2016 10:00	03.04.2016 02:00	40	605	417	0.00	
259	03.04.2016 02:00	04.04.2016 18:00	40	605	417	0.39	0.02
260	05.04.2016 18:00	07.04.2016 07:08	37.1	603	410	0.29	0.01
261	07.04.2016 07:08	08.04.2016 20:17	37.1	603	410	0.44	0.02
262	08.04.2016 20:17	10.04.2016 09:25	37.1	603	410	0.46	0.03

Table S2. Total and species-specific planktonic foraminifera shell fluxes (# m^{-2} d^{-1}).

Sample	Starts time	End time	Bottom-depth (m)	Trap-depth (m)	Total PF	*T. clarkei*	*T. quinqueloba*	*G. ruber*	*G. glutinata*	*G. rubescens + tenellus*	*D. anfracta*	*G. siphonifera*	*G. calida*	*O. universa*	*Unidentified*
1	17.03.2015 18:00	18.03.2015 18:00	604	405	6804	3223	597	239	199	477	159	80	597	0	1233
2	19.03.2015 18:00	20.03.2015 06:00	604	405	657	408	30	30	50	10	0	10	30	0	90
3	20.03.2015 18:00	21.03.2015 06:00	604	405	686	448	50	20	20	20	0	10	40	0	80
4	21.03.2015 18:00	22.03.2015 06:00	604	405	1058	684	56	16	16	80	8	32	64	8	95
5	25.03.2015 06:00	26.03.2015 18:00	603	405	928	796	0	0	0	133	0	0	0	0	0
6	28.03.2015 06:00	29.03.2015 18:00	603	405	928	265	0	398	133	133	0	0	0	0	0
7	31.03.2015 06:00	01.04.2015 18:00	603	405	3846	2210	398	265	88	177	0	44	0	0	663
8	03.04.2015 06:00	04.04.2015 18:00	603	405	15120	7560	2653	928	663	663	398	133	265	0	1857
9	06.04.2015 06:00	07.04.2015 18:00	603	405	11041	7295	1492	564	199	298	0	265	99	464	365
10	09.04.2015 06:00	10.04.2015 18:00	603	405	4358	2141	360	474	152	171	76	284	189	133	379
11	12.04.2015 06:00	13.04.2015 18:00	603	405	1233	548	128	111	71	35	13	93	53	84	97
12	15.04.2015 06:00	16.04.2015 18:00	603	405	3329	1618	398	318	172	146	40	40	106	53	438
13	18.04.2015 06:00	19.04.2015 18:00	603	405	641	345	90	33	53	23	8	17	11	8	53
14	21.04.2015 06:00	22.04.2015 18:00	603	405	632	309	75	35	49	13	0	13	13	40	84
15	26.04.2015 18:00	28.04.2015 06:00	603	398	1956	926	69	69	412	34	0	0	103	0	343
16	29.04.2015 18:00	01.05.2015 06:00	603	398	0	0	0	0	0	0	0	0	0	0	0
17	02.05.2015 18:00	04.05.2015 06:00	603	398	89	45	0	0	0	0	0	0	0	0	45
18	04.05.2015 06:00	05.05.2015 18:00	603	398	0	0	0	0	0	0	0	0	0	0	0
19	05.05.2015 18:00	07.05.2015 06:00	603	398	48	0	0	0	48	0	0	0	0	0	0
20	07.05.2015 06:00	08.05.2015 18:00	603	398	0	0	0	0	0	0	0	0	0	0	0
21	08.05.2015 18:00	10.05.2015 06:00	603	398	90	60	15	0	15	0	0	0	0	0	0
22	10.05.2015 06:00	11.05.2015 18:00	603	398	1271	913	36	36	179	18	54	0	18	18	0
23	11.05.2015 18:00	13.05.2015 06:00	603	398	1833	611	133	80	319	0	27	0	186	0	478
24	13.05.2015 06:00	14.05.2015 18:00	603	398	87	58	0	0	0	0	0	0	0	0	29
25	14.05.2015 18:00	16.05.2015 06:00	603	398	5895	3887	319	64	1179	64	64	0	159	32	127
26	17.05.2015 18:00	19.05.2015 06:00	603	398	4013	2905	200	127	436	109	54	73	18	54	36
27	20.05.2015 18:00	22.05.2015 06:00	603	398	163	93	0	0	0	23	0	0	0	0	47
28	22.05.2015 06:00	23.05.2015 18:00	603	398	0	0	0	0	0	0	0	0	0	0	0
29	23.05.2015 18:00	25.05.2015 06:00	603	398	162	61	0	0	20	0	0	0	0	0	81
30	26.05.2015 18:00	28.05.2015 06:00	603	398	39	26	0	0	0	0	0	0	0	0	13
31	02.06.2015 18:00	04.06.2015 06:00	606	415	424	252	13	13	27	40	0	0	27	0	53
32	05.06.2015 18:00	07.06.2015 06:00	606	415	3006	1481	199	420	44	111	287	44	177	22	221
33	08.06.2015 18:00	10.06.2015 06:00	606	415	2096	1393	186	106	13	172	66	27	40	0	93
34	11.06.2015 18:00	13.06.2015 06:00	606	415	6499	3979	743	769	0	584	27	27	159	0	212
35	14.06.2015 18:00	16.06.2015 06:00	606	415	975	676	73	60	7	93	13	7	27	0	20
36	17.06.2015 18:00	19.06.2015 06:00	606	415	955	650	20	86	13	33	0	7	46	7	93
37	20.06.2015 18:00	22.06.2015 06:00	606	415	729	424	46	80	20	60	20	13	20	7	40
38	23.06.2015 18:00	25.06.2015 06:00	606	415	1048	729	93	13	13	80	13	0	27	0	80
39	26.06.2015 18:00	28.06.2015 06:00	606	415	371	225	0	27	7	80	0	0	0	0	33
40	29.06.2015 18:00	01.07.2015 06:00	606	415	537	305	27	53	20	20	0	13	33	7	60

Table S2. Cont.

Sample	Starts time	End time	Bottom-depth (m)	Trap-depth (m)	Total PF	*T. clarkei*	*T. quinqueloba*	*G. ruber*	*G. glutinata*	*G. rubescens + tenellus*	*D. anfracta*	*G. siphonifera*	*G. calida*	*O. universa*	*Unidentified*
41	02.07.2015 18:00	04.07.2015 06:00	606	415	1207	902	53	80	0	27	0	13	13	13	106
42	05.07.2015 18:00	07.07.2015 06:00	608	408	206	133	0	66	7	0	0	0	0	0	0
43	11.07.2015 18:00	13.07.2015 06:00	608	408	1432	875	53	265	0	106	13	0	27	0	93
44	14.07.2015 18:00	16.07.2015 06:00	608	408	862	630	0	66	133	33	0	0	0	0	0
45	17.07.2015 18:00	19.07.2015 06:00	608	408	477	265	13	80	13	53	0	0	0	0	53
46	20.07.2015 18:00	22.07.2015 06:00	608	408	1724	1406	106	106	27	80	0	0	0	0	0
47	23.07.2015 18:00	25.07.2015 06:00	608	408	1963	1114	186	292	27	133	0	27	0	0	186
48	26.07.2015 18:00	28.07.2015 06:00	608	408	451	279	66	80	13	0	13	0	0	0	0
49	29.07.2015 18:00	31.07.2015 06:00	608	408	995	623	119	93	53	66	0	0	40	0	0
50	01.08.2015 18:00	03.08.2015 06:00	608	408	418	358	7	20	20	0	0	0	13	0	0
51	04.08.2015 18:00	06.08.2015 06:00	608	408	358	225	49	40	9	9	0	4	0	0	22
52	11.08.2015 18:00	13.08.2015 06:00	604	412	544	133	40	265	22	18	0	13	22	9	22
53	14.08.2015 18:00	16.08.2015 06:00	604	412	398	212	27	53	27	80	0	0	0	0	0
54	17.08.2015 18:00	19.08.2015 06:00	604	412	796	424	80	159	27	53	0	0	27	0	27
55	20.08.2015 18:00	22.08.2015 06:00	604	412	955	358	159	199	0	40	0	0	53	0	146
56	23.08.2015 18:00	25.08.2015 06:00	604	412	690	305	27	172	13	80	27	0	53	0	13
57	26.08.2015 06:00	27.08.2015 06:00	604	412	448	239	80	90	0	0	0	40	0	0	0
58	28.08.2015 06:00	29.08.2015 06:00	604	412	1711	756	378	358	40	80	0	40	40	0	20
59	30.08.2015 06:00	31.08.2015 06:00	604	412	1631	935	199	338	60	0	0	0	40	0	60
60	01.09.2015 06:00	02.09.2015 06:00	604	412	875	458	40	259	99	0	0	20	0	0	0
61	03.09.2015 06:00	04.09.2015 06:00	604	412	1393	935	129	159	0	90	0	40	0	0	40
62	05.09.2015 06:00	06.09.2015 06:00	604	412	2268	1393	199	398	0	119	60	40	20	0	40
63	10.09.2015 10:00	11.09.2015 18:00	608	408	2268	1552	328	209	30	0	0	0	0	0	149
64	13.09.2015 02:00	14.09.2015 10:00	608	408	2350	1567	149	336	0	75	0	0	37	37	149
65	15.09.2015 18:00	17.09.2015 02:00	608	408	1552	895	119	269	60	90	0	0	0	0	119
66	18.09.2015 10:00	19.09.2015 18:00	608	408	836	537	149	90	0	0	0	0	0	0	60
67	22.09.2015 10:00	23.09.2015 18:00	608	408	1368	821	99	323	0	25	0	0	25	0	75
68	25.09.2015 02:00	26.09.2015 10:00	608	408	905	347	196	272	0	60	0	30	0	0	0
69	27.09.2015 18:00	29.09.2015 02:00	608	408	1641	767	490	298	0	21	0	21	21	0	21
70	01.10.2015 18:00	03.10.2015 02:00	608	408	237	108	22	108	0	0	0	0	0	0	0
71	04.10.2015 10:00	05.10.2015 18:00	608	408	71	0	0	71	0	0	0	0	0	0	0
72	07.10.2015 18:00	08.10.2015 22:00	600	411	77	34	0	43	0	0	0	0	0	0	0
73	10.10.2015 02:00	11.10.2015 06:00	600	411	584	175	29	204	0	29	0	0	0	0	146
74	12.10.2015 10:00	13.10.2015 14:00	600	411	19	19	0	0	0	0	0	0	0	0	0
75	13.10.2015 14:00	14.10.2015 18:00	600	411	864	555	49	25	37	37	62	0	37	12	49
76	14.10.2015 18:00	15.10.2015 22:00	600	411	0	0	0	0	0	0	0	0	0	0	0
77	17.10.2015 02:00	18.10.2015 06:00	600	411	0	0	0	0	0	0	0	0	0	0	0
78	19.10.2015 10:00	20.10.2015 14:00	600	411	86	43	0	0	0	0	0	0	0	14	29
79	21.10.2015 18:00	22.10.2015 22:00	600	411	805	560	0	245	0	0	0	0	0	0	0
80	24.10.2015 02:00	25.10.2015 06:00	600	411	1654	792	303	326	47	93	0	0	93	0	0

Table S2. Cont.

Sample	Starts time	End time	Bottom-depth (m)	Trap-depth (m)	Total PF	*T. clarkei*	*T. quinqueloba*	*G. ruber*	*G. glutinata*	*G. rubescens + tenellus*	*D. anfracta*	*G. siphonifera*	*G. calida*	*O. universa*	*Unidentified*
81	26.10.2015 10:00	27.10.2015 14:00	600	411	3956	2230	288	1223	216	0	0	0	0	0	0
82	27.10.2015 14:00	28.10.2015 18:00	600	411	3427	1980	685	609	0	0	0	0	152	0	0
83	28.10.2015 18:00	29.10.2015 22:00	600	411	3741	1370	105	2002	53	105	0	53	53	0	0
84	31.10.2015 02:00	01.11.2015 06:00	600	411	1891	779	74	927	0	74	0	0	37	0	0
85	02.11.2015 18:00	04.11.2015 02:00	605	403	1461	643	117	351	117	58	0	0	58	0	117
86	05.11.2015 10:00	06.11.2015 18:00	605	403	7174	1843	132	4475	263	197	0	66	0	0	197
87	08.11.2015 02:00	09.11.2015 10:00	605	403	1832	1274	0	239	80	0	0	0	0	0	239
88	09.11.2015 10:00	10.11.2015 18:00	605	403	1611	970	164	388	60	0	0	0	0	0	30
89	10.11.2015 18:00	12.11.2015 02:00	605	403	3577	2429	309	442	221	88	0	0	88	0	0
90	12.11.2015 02:00	13.11.2015 10:00	605	403	2802	2388	92	276	46	0	0	0	0	0	0
91	14.11.2015 18:00	16.11.2015 02:00	605	403	610	450	32	0	64	32	0	0	0	0	32
92	17.11.2015 10:00	18.11.2015 18:00	605	403	3017	1584	189	415	151	226	0	0	339	0	113
93	20.11.2015 02:00	21.11.2015 10:00	605	403	3092	2329	267	305	76	76	0	0	0	0	38
94	22.11.2015 18:00	24.11.2015 02:00	605	403	3342	2641	209	149	104	134	0	0	90	0	15
95	24.11.2015 02:00	25.11.2015 10:00	605	403	8973	5731	984	810	289	347	116	58	289	0	347
96	25.11.2015 10:00	26.11.2015 18:00	605	403	9342	6197	1156	1295	139	370	92	0	92	0	0
97	26.11.2015 18:00	28.11.2015 02:00	605	403	3282	2387	398	298	99	0	0	0	0	99	0
98	29.11.2015 10:00	30.11.2015 18:00	605	403	872	537	101	67	0	0	0	0	34	0	134
99	02.12.2015 18:00	04.12.2015 00:00	613	408	1432	1061	0	212	106	0	0	0	0	0	53
100	05.12.2015 06:00	06.12.2015 12:00	613	408	5109	2785	318	1066	95	80	589	80	95	0	0
101	07.12.2015 18:00	09.12.2015 00:00	613	408	4987	1485	637	1114	159	53	584	106	371	53	424
102	11.12.2015 12:00	12.12.2015 18:00	613	408	2865	1146	191	605	64	64	684	48	64	0	0
103	14.12.2015 00:00	15.12.2015 06:00	613	408	9284	4456	584	1751	106	1167	477	53	371	0	318
104	16.12.2015 12:00	17.12.2015 18:00	613	408	4350	2016	477	424	477	159	531	0	106	0	159
105	19.12.2015 00:00	20.12.2015 06:00	613	408	3183	1846	223	286	32	223	446	0	95	0	32
106	21.12.2015 12:00	22.12.2015 18:00	613	408	3621	2745	279	119	40	0	398	0	0	0	40
107	22.12.2015 18:00	24.12.2015 00:00	613	408	5332	2905	716	279	398	40	358	0	80	0	557
108	24.12.2015 00:00	25.12.2015 06:00	613	408	2175	1167	106	318	212	53	212	0	106	0	0
109	25.12.2015 06:00	26.12.2015 12:00	613	408	2865	1167	690	159	159	106	477	0	53	0	53
110	27.12.2015 18:00	29.12.2015 00:00	613	408	1273	849	0	159	159	0	106	0	0	0	0
111	30.12.2015 18:00	01.01.2016 06:00	612	417	265	0	0	133	0	0	0	0	0	0	133
112	02.01.2016 18:00	04.01.2016 06:00	612	417	1459	398	0	928	0	0	0	0	133	0	0
113	05.01.2016 18:00	07.01.2016 06:00	612	417	1127	729	66	0	0	66	0	0	133	0	133
114	08.01.2016 18:00	10.01.2016 06:00	612	417	796	663	0	0	0	133	0	0	0	0	0
115	11.01.2016 18:00	13.01.2016 06:00	612	417	2122	1393	332	133	0	66	66	66	66	0	0
116	14.01.2016 18:00	16.01.2016 06:00	612	417	597	398	133	0	0	66	0	0	0	0	0
117	17.01.2016 18:00	19.01.2016 06:00	612	417	7560	5438	1459	133	0	133	133	133	133	0	0
118	20.01.2016 18:00	22.01.2016 06:00	612	417	4775	2122	663	265	265	265	796	0	265	0	133
119	23.01.2016 18:00	25.01.2016 06:00	612	417	1989	1061	133	133	0	265	133	0	133	0	133
120	26.01.2016 18:00	28.01.2016 06:00	612	417	2034	1149	88	354	88	133	133	44	44	0	0

Table S2. Cont.

Sample	Starts time	End time	Bottom-depth (m)	Trap-depth (m)	Total PF	*T. clarkei*	*T. quinqueloba*	*G. ruber*	*G. glutinata*	*G. rubescens + tenellus*	*D. anfracta*	*G. siphonifera*	*G. calida*	*O. universa*	*Unidentified*
121	29.01.2016 18:00	31.01.2016 06:00	612	417	7029	4377	597	332	199	332	531	66	199	0	398
122	01.02.2016 18:00	03.02.2016 01:00	600	403	4852	2618	77	1540	77	385	0	0	154	0	0
123	04.02.2016 08:00	05.02.2016 15:00	600	403	5468	2926	0	1386	77	231	77	0	154	0	616
124	06.02.2016 22:00	08.02.2016 05:00	600	403	5904	4107	308	770	0	411	0	0	154	0	154
125	09.02.2016 12:00	10.02.2016 19:00	600	403	2670	1592	205	103	0	103	103	154	154	0	257
126	12.02.2016 02:00	13.02.2016 09:00	600	403	11937	8240	1155	847	0	1078	0	77	462	0	77
127	14.02.2016 16:00	15.02.2016 23:00	600	403	17135	13554	1386	655	39	578	77	154	77	0	616
128	17.02.2016 06:00	18.02.2016 13:00	600	403	282	185	26	5	0	31	10	5	15	5	0
129	19.02.2016 20:00	21.02.2016 03:00	600	403	323	185	54	15	0	39	0	15	15	0	0
130	22.02.2016 10:00	23.02.2016 17:00	600	403	298	154	31	41	15	10	10	5	15	5	10
131	25.02.2016 00:00	26.02.2016 07:00	600	403	647	185	154	62	0	31	154	31	0	31	0
132	02.03.2016 10:00	04.03.2016 02:00	605	417	27295	16552	2268	2109	2348	1671	517	597	279	318	637
133	05.03.2016 18:00	07.03.2016 10:00	605	417	17487	12474	1890	219	736	736	259	298	418	20	438
134	09.03.2016 02:00	10.03.2016 18:00	605	417	21145	14273	3257	119	819	648	460	767	273	17	512
135	12.03.2016 10:00	14.03.2016 02:00	605	417	4775	2149	1114	119	199	358	0	199	159	80	398
136	17.03.2016 10:00	19.03.2016 02:00	605	417	1206	609	406	12	36	48	24	12	12	0	48
137	03.04.2016 02:00	04.04.2016 18:00	605	417	1301	752	155	36	155	72	60	48	12	0	12
138	08.04.2016 20:17	10.04.2016 12:17	603	410	2172	812	633	322	191	60	12	48	36	0	60

Table S3. Correlation coefficients of planktonic foraminifera species flux, moon illumination, SST, Chl-*a*, particulate bulk and POC fluxes.

	**Moon illumination**	***T. clarkei***	***T. quinqueloba***	***G. ruber***	***G. glutinata***	***G. rubescens & tenellus***	***D. anfracta***	***G. siphonifera***	***G. calida***	***O. universa***	**Unidentified PF**	**SST**	**Chl-*a***	**Bulk flux**	**POC flux**
**Moon illumination**	1.00	-0.09	0.00	-0.04	-0.07	-0.11	0.01	-0.10	-0.15	0.06	-0.02	-0.05	0.07	0.04	0.15
T. clarkei	-0.09	1.00	0.90	0.41	0.76	0.88	0.51	0.84	0.68	0.52	0.60	-0.33	0.25	0.08	0.34
T. quinqueloba	0.00	0.90	1.00	0.29	0.67	0.77	0.53	0.85	0.66	0.49	0.68	-0.32	0.27	0.10	0.41
G. ruber	-0.04	0.41	0.29	1.00	0.38	0.50	0.29	0.28	0.35	0.27	0.31	-0.02	0.11	0.22	0.31
G. glutinata	-0.07	0.76	0.67	0.38	1.00	0.75	0.50	0.74	0.48	0.60	0.48	-0.26	0.28	0.01	0.36
G. rubescens & tenellus	-0.11	0.88	0.77	0.50	0.75	1.00	0.52	0.73	0.71	0.51	0.57	-0.35	0.24	0.09	0.39
D. anfracta	0.01	0.51	0.53	0.29	0.50	0.52	1.00	0.47	0.58	0.23	0.37	-0.35	0.23	0.18	0.35
G. siphonifera	-0.10	0.84	0.85	0.28	0.74	0.73	0.47	1.00	0.56	0.62	0.50	-0.32	0.31	-0.03	0.25
G. calida	-0.15	0.68	0.66	0.35	0.48	0.71	0.58	0.56	1.00	0.27	0.59	-0.38	0.19	0.15	0.31
O. universa	0.06	0.52	0.49	0.27	0.60	0.51	0.23	0.62	0.27	1.00	0.36	-0.22	0.20	-0.04	0.20
**Unidentified PF**	-0.02	0.60	0.68	0.31	0.48	0.57	0.37	0.50	0.59	0.36	1.00	-0.32	0.12	0.13	0.48
**SST**	-0.05	-0.33	-0.32	-0.02	-0.26	-0.35	-0.35	-0.32	-0.38	-0.22	-0.32	1.00	-0.56	-0.23	-0.32
**Chl-*a***	0.07	0.25	0.27	0.11	0.28	0.24	0.23	0.31	0.19	0.20	0.12	-0.56	1.00	0.16	0.21
**Bulk flux**	0.04	0.08	0.10	0.22	0.01	0.09	0.18	-0.03	0.15	-0.04	0.13	-0.23	0.16	1.00	0.72
**POC flux**	0.15	0.34	0.41	0.31	0.36	0.39	0.35	0.25	0.31	0.20	0.48	-0.32	0.21	0.72	1.00
